# Bismuth release from endodontic materials: in vivo analysis using Wistar rats

**DOI:** 10.1038/s41598-023-36690-4

**Published:** 2023-06-15

**Authors:** M. A. Marciano, L. E. Pelepenko, T. M. Francati, T. B. M. Antunes, A. C. P. Janini, J. J. R. Rohwedder, R. M. Shelton, J. Camilleri

**Affiliations:** 1 Piracicaba Dental School of Dentistry, University of Campinas, Piracicaba, Brazil; 2 Institute of Chemistry, University of Campinas, Campinas, Brazil; 3 School of Dentistry, College of Medical and Dental Sciences, University of Birmingham, 5, Mill Pool Way, Edgbaston, Birmingham, B5 7EG UK

**Keywords:** Dental materials, Endodontics, Restorative dentistry

## Abstract

Calcium silicate-based materials are used to block the communication between the root canal and the periodontal ligament space. This brings the materials into contact with tissues and the potential for local and systemic elemental release and movement. The aim of the study was to evaluate the elemental release of bismuth from ProRoot MTA in contact with connective tissues after 30 and 180 days as well as any accumulation in peripheral organs using an animal model. Tricalcium silicate and hydroxyapatite containing 20% bismuth oxide (HAp-Bi) were used as controls. The null hypothesis was that bismuth migrates from tricalcium silicate-based materials when associated with silicon. The materials were examined using scanning electron microscopy, energy dispersive spectroscopy (SEM/EDS) and X-ray diffraction prior to implantation as well as using SEM/EDS, micro X-ray fluorescence and Raman spectroscopy after implantation to assess elemental presence in surrounding tissues. Histological analysis was used to evaluate the changes in tissue architecture and inductively coupled plasma mass spectrometry (ICP-MS) was used to investigate the elemental deposition. For the systemic investigation, routine blood analysis was performed and organs were obtained to evaluate the presence of bismuth and silicon using ICP-MS after acid digestion. In the histological analysis of the implantation sites, macrophages and multinucleated giant cells could be observed after 30 days which after 180 days became a chronic infiltrate; although, no major differences were identified in red and white blood cell analyses and biochemical tests. Implantation altered the materials as observed in the Raman analysis and bismuth was detected both locally and within kidney samples after both periods of analysis, indicating the potential for accumulation of bismuth in this organ. Smaller amounts of bismuth than observed in the kidney were also detected in blood, liver and brain for the ProRoot MTA and HAp-Bi after 180 days. Bismuth was released from the ProRoot MTA locally and was detected systemically and in samples without silicon; thus, the null hypothesis was rejected. The bismuth release demonstrated that this element accumulated both locally and systemically, mainly in the kidneys in comparison with brain and liver regardless of the material base.

## Introduction

The management of endodontic treatment failure and procedural errors resulting in a communication between the root canal and periodontal tissues requires the placement of a material plug. Mineral trioxide aggregate (MTA) and other tricalcium silicate-based cements are the materials of choice for surgical and reparative procedures in endodontics. Over the years, MTA has been used for vital pulp treatments in paediatric patients^1,2^ and also used to manage the permanent dentition^3–6^. It is also used for sealing of root perforations^7^, apexification^8^, surgical procedures^9–13^ and as a cervical barrier material in dental revascularization procedures^14,15^. For all the clinical procedures the material comes into contact with the host tissues.

ProRoot MTA (Denstsply, Tulsa, OK, USA) is the most well established mineral trioxide aggregate that has been used and researched extensively since its development^16^ and first patent registration^17^ in 1995. It is composed of Portland cement which in turn is composed of tricalcium silicate, dicalcium silicate, tricalcium aluminate and calcium sulphate hemihydrate^18,19^ and bismuth oxide as the radiopacifier^19^. When mixed with water, cement hydration occurs resulting in the formation of calcium silicate hydrate and calcium hydroxide from the reaction of tricalcium silicate with water^20^ and the bismuth oxide supposedly acting as an inert filler. Calcium and hydroxyl ions are released over time^21,22^ leading to an increased alkalinity^23,24^.

Although bismuth oxide should be inert and act only as radiopacifier, it has been reported to displace the silicon in tricalcium silicate^22^. Elemental release of bismuth from the material into the tooth structure^25^ has also shown the instability of bismuth within MTA. Other elements such as silicon are also released from the material into dentine^25–27^ and both bismuth and silicon from MTA have been identified in adjacent connective tissues^28^.

The elemental release has a number of implications. Bismuth oxide in MTA is very reactive when in contact with collagen in dentine^29^, sodium hypochlorite^25,30^ and other solutions used in endodontics^31^ as well as in contact with blood^28^. It forms metastable bismuth complexes that are black and thus discolour the material and adjacent tissues. Although silicon release from the material into tissues has been reported^27,28,32^, there is limited evidence regarding the implications for the host tissues.

The Globally Harmonized System is a standard system for identifying hazardous properties throughout the world. Depending on the world region the material is used in^33–35^, safety data sheets show it as safe to use or a chemwatch hazard considering the ratings of chronic and body contact as a highly hazardous chemical^36^. When classified as a hazardous chemical, it is stated that the absorbed bismuth salts permeate the body fluids and tissues and are excreted mainly in urine, but some bismuth is retained in tissues and that there may be a blue line (“bismuth line”) on the gums years later^36^. This safety data sheet also states that a potential environmental toxicity and accumulation may be present as a danger indicating that research on systemic toxicity is warranted.

Systemic release of bismuth is a serious concern due to its potential cytotoxicity, as bismuth has been associated with toxic effects in human pulp cells^37^ and also in contact with liver, kidney, intestinal and lung cells of experimental animals^38,39^. The material contact time-related release pattern with biological tissues is unknown. This may be a concern, since after its clinical use this type of material is intended to remain in situ for an extended period and in many cases, throughout the patient's life.

Previous studies reported the association between encephalopathy and the administration of bismuth iodoform paraffin pastes^40–42^, mineral water^43^ and bismuth salts^44–55^ indicating that absorbed bismuth is able to cross the blood–brain barrier after its administration potentially resulting in neurological effects^42,56–58^. The bismuth that is circulating systemically is subject to a detoxification process where the last step is lysosomal storage of this heavy metal^59,60^.

The aims of the present study were to assess elemental release of bismuth from ProRoot MTA into connective tissues adjacent to the implantation site; to quantify systemically ProRoot MTA by-products circulating in blood and the potential uptake by different organs after two different periods of implantation. It was postulated that bismuth migration may be related to the presence of silicon in tricalcium silicate. Thus the null hypothesis was that bismuth migrates associated with silicon.

## Materials and methods

The present study was performed using an animal model (ethical approval for animals in research, CEUA process number 5034-1/2018). The sample size was calculated using Gpower 3.0 software^61^, considering a power test of 0.8. A total of 66 (n = 10), 12-week old male rats (*Rattus norvegicus*, Albinus, Wistar) weighing 369.6 ± 31.8 g with no significant difference between them (*p* = 0.073) were used. All procedures performed followed the NIH Guide for Care and Use of Laboratory Animals guidelines^62^ and ARRIVE guidelines for animal testing. Ethical approval was obtained form the Piraacicaba Dental School Research Committee.

The following materials were tested:

- ProRoot MTA (Dentsply Tulsa Dental, Tulsa, OK, USA), composed of 80% Portland cement and 20% bismuth oxide^36^; mixed at a water-to-powder ratio of 0.33, following manufacturer’s recommendations (MTA).
- Tricalcium silicate (Mineral Research Processing, Meyzieu, France); mixed at a water-to-powder ratio of 0.35 (TCS).
- Hydroxyapatite (Mineral Research Processing, Meyzieu, France) containing 20% of bismuth oxide (Merck KGaA, Darmstadt, Germany); mixed at a water-to-powder ratio of 0.25 (HAp-Bi).

**Figure 1:**
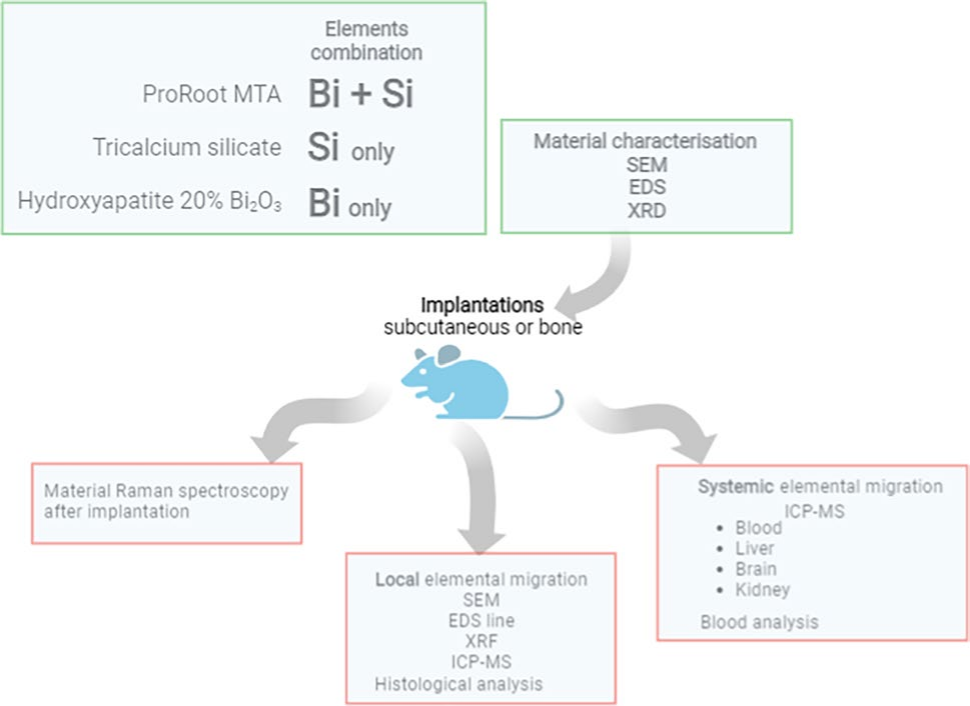
Schematic diagram showing the testing undertaken.

To investigate the potential bismuth migration associated with silicon the material selection included MTA, whereas TCS was included containing silicon only and HAp-Bi containing bismuth, but without silicon. A schematic diagram showing the testing undertaken is shown in Fig. 1.

### Material characterization

A set of unhydrated and hydrated material samples were examined using scanning electron microscopy (SEM), energy-dispersive X-ray spectroscopy (EDS) and phase analysis assessed using X-ray diffraction (XRD) prior to the animal implantation.

#### Powder characterization

For unhydrated sample analysis, powders were placed directly onto adhesive carbon tape on aluminium stubs without coating. Scanning electron micrographs (SEM; EVO MA10, Carl Zeiss Ltd., Cambridge, UK) were obtained in backscatter mode at different magnifications and energy dispersive spectroscopy (EDS) was performed to obtain the elemental analysis.

Phase identification of the unhydrated powders was carried out using an X-ray diffractometer (Bruker D8 Advance, Bruker Corp., Billerica, MA, USA). The powders were directly placed in the sample holder and XRD pattern peaks were obtained with a CuKa radiation at 40 mA and 45 kV, set to rotate between 10 and 60°, with a step size of 0.2° and a coupled 2θ intensity factor. Phase identification of the ‘xy’ files was undertaken using search-match software (DIFFRAC.EVA, Bruker Corp., Billerica, MA, USA) based on the crystallography open database (COD-Inorg database) for the component peak matching.

#### Characterization of set materials

For SEM/EDS analysis of the hydrated samples, freshly mixed materials were placed in 10 × 2 mm circular moulds and allowed to set for 24 h at 37 °C in a 95% humidity chamber (Memmert GmbH, Büchenbach, Germany) and vacuum desiccated for another 24 h. The samples were embedded in resin (EpoFix, Struers, Ballerup, Denmark) and ground using progressively finer discs using water coolant (Piano 220, 500 and 1200) followed by polishing using cloths (Largo, Dac, Nap and Chem) with increasing fineness of diamond impregnated solution in an automatic polishing machine (Struers Tegramin-25, Ballerup, Denmark). The embedded samples were dried in a vacuum desiccator, mounted on aluminum stubs and gold coated (Emitech K550X sputter coater, Heathfield East Sussex, UK). SEM photomicrographs were obtained in backscatter mode at different magnifications and coupled EDS used to obtain the spectra in representative areas of the material surface at 1500 × magnification.

For the X-ray diffraction analysis, freshly mixed samples were prepared as previously described for the SEM/EDS analysis and after vacuum desiccation samples were crushed using an agate mortar and pestle into a fine powder for the bulk phase identification. The XRD peaks and COD matching were obtained using the same settings as described above for powder characterization.

### Assessment of in vivo elemental dispersion and tissue inflammatory response

For the implantation procedures, the animals were randomly divided in two groups (n = 33 each) according to the experimental periods of 30 and 180 days of implantation. Subcutaneous dorsal connective tissue and tibial bone were targeted for each tested material/period (n = 10) and for the controls (n = 6) no surgical procedures were performed. The test animals were anaesthetized with a combination of ketamine (90 mg/kg) and xylazine (10 mg/kg) (Vet Brands Int, Miramar, Florida, USA).

In the subcutaneous implantation group, the dorsal region was trichotomized and disinfected with 1% iodine. A longitudinal incision was made using a #15 scalpel blade and freshly mixed material samples were placed subcutaneously. Materials were made in a 4 mm diameter × 2 mm thickness mould before placing in intimate contact with the connective tissues^28,63^. Each animal received two similar implants of the same material weighing between 0.040 and 0.055 g at least 10 mm from the incision. Following sample placement the incision borders were reapproximated and sutured with 4–0 silk suture. For the bone implantation group, the tibial region was trichotomized and disinfected with 1% iodine. A longitudinal incision over the tibial bone was made using a #15 scalpel blade. After exposing the bone by gently reflecting overlying tissues, two 4 mm × 2 mm circular cavities 10 mm distant from each other were gradually prepared with a calibrated 2-mm-depth 1016 diamond-bur (KG Sorensen, Sao Paulo, Brazil) under constant irrigation with sterile 0.9% saline solution. The diameter was measured using a calibrated periodontal probe. The same freshly mixed material was placed in both cavities in each animal and incisions were sutured using 4–0 silk. Dipyrone was intramuscularly administered (100 mg/kg) immediately after the procedures. The animals were kept in a controlled room temperature of 20 ± 1 °C with water and food ad libitum during the experimental period and after 30 and 180 days all animals were euthanazed with an overdose of anaesthetic. The samples were retrieved and processed as indicated in Fig. 1.

#### Animal-to-human age equivalency

Using a previously reported^64^ equivalent to human age, the animals' age used in the present study was converted to enable comparison.

#### Local elemental release

For the SEM/EDS material-tissue interface analysis, the first material implant along with the adjacent subcutaneous or bone tissues were retrieved using a scalpel blade and scissors, fixed in 70% ethanol, dried with ascending grades of ethanol, critical-point dried, mounted in resin-stubs, polished and carbon-coated. SEM (JEOL, JSM-IT300, Akishima, Tokyo, Japan) photomicrographs in secondary electron mode were obtained and elemental mapping performed using the line EDS tool to determine the distribution of bismuth, silicon, calcium and phosphorus along the material-tissue interface. Connective tissues not exposed to materials were used as controls.

The material-tissue interface samples were also analysed using a benchtop m-XRF (Orbis PC EDAX, United States) with a Rh anode using 50 W maximum power. To optimize the analysis for bismuth, a 100 mm rhodium filter was selected for the analysis and detection was carried out using a 30 mm^2^ silicon drift detector (140 eV FWHM at the 5.9 keV Mn-Ka line) pre-set to an X-ray beam size of 30 µm, a tube current of 500 µA, voltage of 45 kV, dwell time of 120 s, mapping 32 points without vacuum.

Tissues samples adjacent to the second material implant were collected and immediately stored at – 20 °C until the elemental ICP-MS analysis was carried out^65^. A microwave digestion system (Milestone, Shelton, CT, USA) was used for the acid digestions and 6 ml 20% P.A. nitric acid (Sigma Aldrich—Merck, Germany) redistilled by a sub-boiling process and 2 ml 30% hydrogen peroxide (Sigma Aldrich—Merck, Germany) were added to each tissue sample for the microwave cycle set to a constant power of 1000 W and pressure of 20 bar for 5 min at 160 °C, 2 min at 160 °C, 5 min at 170 °C and 15 min at 170 °C. Along with each digestion cycle, one blank without sample was obtained using the same cycling conditions. Inductively coupled plasma mass spectrometry (ICP-MS, Optima 8000 Perkin Elmer, Waltham, MA, USA) was used for the elemental quantification of bismuth and silicon. The calibration curves for the ICP-MS analysis were prepared using calibration standards with concentrations of 1000 ± 3 µg/ml prepared in 2% nitric acid (HPS, High Purity Standards, North Charleston, SC, USA) for each assessed element. Mass fractions mean, standard deviation, minimum and maximum values as well as median were expressed in nanograms per gram (ng/g) for each element. The filtered water, feed and wood shavings used for the animals' maintenance were also evaluated with ICP-MS using the same conditions.

#### Systemic elemental release

A volume of 3 ml of blood and 200–300 mg samples of liver, kidneys and brain were collected, aliquoted and immediately stored at − 20 °C until analysis. The acidic digestion and the ICP-MS analysis of the blood and organs samples were performed as previously described for the local tissue elemental analysis.

#### Blood analysis

A sample of 0.5 ml blood was collected and stored in haemogram vials internally coated with anticoagulant for analysis using impedance methods. Erythrocytes, haemoglobin, haematocrit, mean corpuscular volume (MCV), mean corpuscular haemoglobin concentration (MCHC), total blood protein, leukocytes, platelets, segmented neutrophils, eosinophils, lymphocytes and monocytes were assessed in comparison with the control animals. In addition, 1.0 ml blood was stored in aluminum-covered vials for the biochemical colorimetric assessments of plasma bilirubin (total and fractions), alkaline phosphatase (ALP), glutamic-oxalacetic transaminase (GOT), alanine aminotransferase (ALT) and gamma-glutamyl transpeptidase (GGT) for assessment of liver function. Creatinine and urea were also measured from the blood samples to evaluate kidney function. For both analyses, the samples were stored in contact with cooling propylene glycol-based packs and immediately processed. Additionally, the parameter values obtained for the test animals and controls of this study were compared with reference values obtained from previously reported data for Wistar rats^66,67^.

#### Histological assessments

The subcutaneous tissue adherent to the second material implant was retrieved from the animal using a scalpel blade, separated from the material and fixed in 10% formalin for 48 h. Similarly, bone adjacent to the material implant was also separated after retrieval using a cylindrical bur (1090, Ref.: 1801.1090, KG Sorensen, Sao Paulo, Brazil) and decalcified in 17% ethylenediaminetetraacetic acid for 48 h. In sequence, all specimens were washed in water for 24 h, dehydrated in a sequence of alcohols, cleared with xylol, paraffin-embedded and 5 µm thickness sections cut using a microtome (Leica RM 2155, Nussloch, Germany). The samples were stained with haematoxylin and eosin (H&E) for histological analysis. A microscope Leica DM 5000 B (Leica, Milan, Italy) connected to a camera Leica DC 300 F (Leica, Milan, Italy) was used to capture representative images at different magnifications. For each animal, 2 sections of the surrounding tissues in direct contact with the implanted materials were evaluated in 5 different microscopic fields, which were randomly chosen. The inflammatory infiltrate in the subcutaneous tissue and bone were evaluated subjectively and the scores in relation to the extent and intensity were recorded. In addition, the average number of inflammatory cells in the 5 fields were calculated and classified as a score: 0—no cells, absent; 1–< 25 cells, mild; 2–25–125 cells, moderate; and 3–> 125 cells, severe. The scores for inflammation were compared among materials and non-implanted controls samples.

### Material characterization after tissue contact

The second material samples, removed from subcutaneous and bone tissues, were characterised using Raman spectroscopy. The material samples were retrieved, placed inside Eppendorf tubes containing gauze-covered soda lime (Sigma Aldrich—Merck, Germany) and vacuum desiccated for 24 h. Control material samples with the same dimensions—not implanted in the animals—were prepared and allowed to set for 28 days at 37 °C in a 95% humidity chamber and vacuum desiccated for 24 h under the same conditions used for the materials characterization. Raman spectra were obtained using an adjustable laboratory-made spectrometer^68^ with sample excitation from a 785 nm laser beam (Cobolt 08 series 0785-08-11-0500-200; Hubner Photonics, Kassel, Germany) with 20 mW power. The spectrometer was equipped with a 500-mm focal length monochromator (Andor/Oxford SR-500i-C-SIL; Shamrock, Belfast, UK) and a charge-coupled device camera (Andor/Oxford iDUS 416 -DU416A-LDC-DD; Shamrock). The spectral resolution was approximately 2 cm^−1^ and the range was set from 0 to 1300 cm^−1^. For comparisons, data were processed by individually adjusting the intensity axis for each sample with the same Raman shift intervals.

### Statistical analysis

GraphPad software (Version 9.3.1) was used for statistical analysis. Shapiro–Wilk test was used for parametric assessments followed by ANOVA with Tukey’s multiple comparisons test (for parametric data) or the Kruskal–Wallis with Dunn's multiple comparisons test (for non-parametric data) to assess differences between the groups. For independent comparisons between two groups, an unpaired T-test or two-tailed Mann–Whitney test was used according to the data distribution. All statistical tests were performed at a significance level of 5% (*p* < 0.05).

## Results

### Material characterization

#### Powder characterization

**Figure 2:**
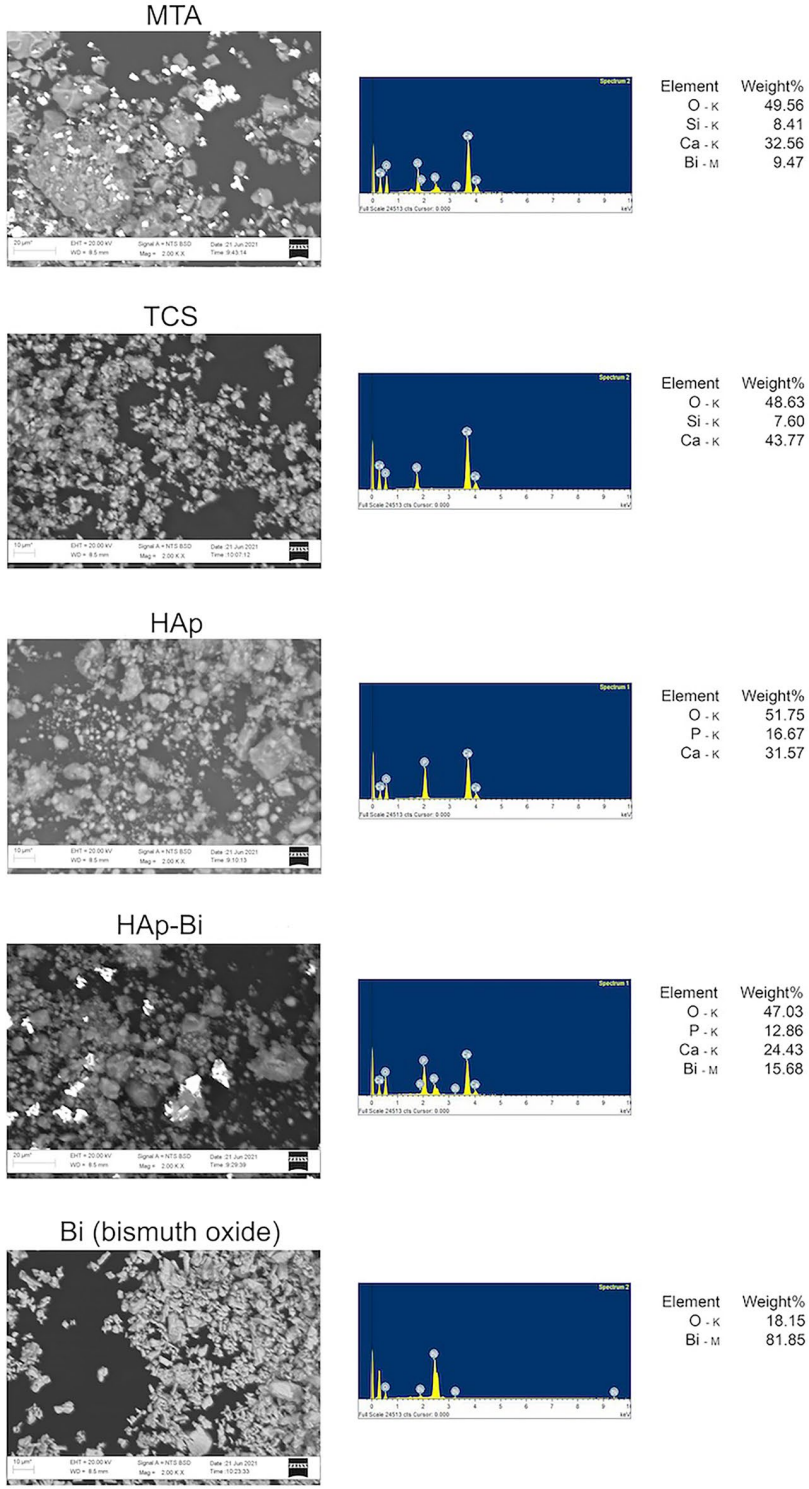

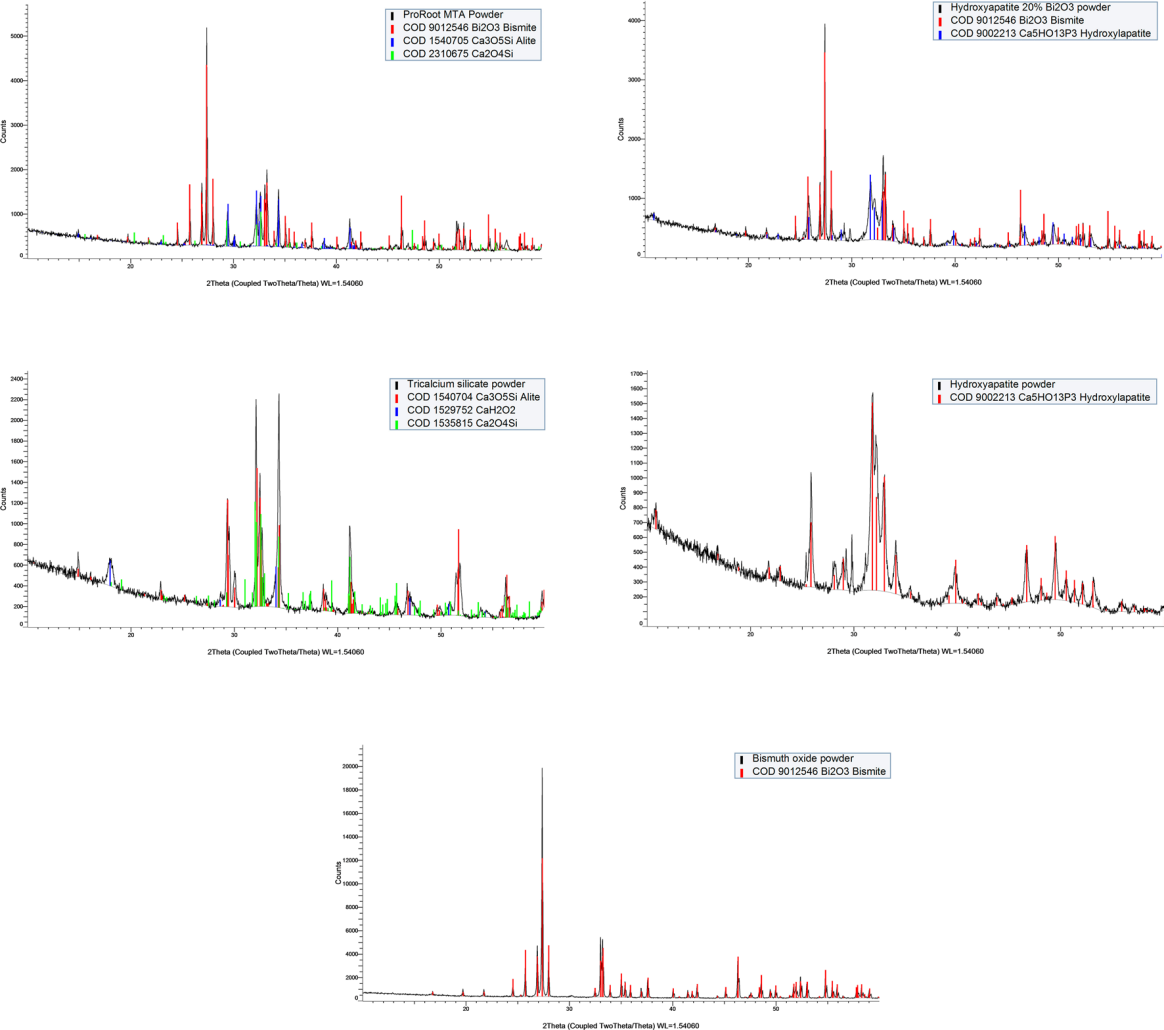
(**a**) SEM/EDS and (**b**) XRD characterization of the material powders.

SEM and EDS analysis of the unhydrated materials is shown in Fig. 2a. The MTA powder was mainly composed of calcium and silicon, corresponding with the cement particles and different sizes of bismuth-containing particles were also detected. The TCS was found to be composed of calcium and silicon only and similar sized particles were observed. The pure hydroxyapatite was rich in calcium and phosphorus, whereas Hap-Bi exhibited elongated bismuth particles interspersed within the less radiopaque particles. X-ray diffractograms of the unhydrated materials are shown in Fig. 2b. MTA powder exhibited peaks of bismuth oxide (Bi_2_O_3_) (COD 9,012,546), tricalcium silicate in alite crystalline form (Ca_3_O_5_Si) (COD 1,540,705) and dicalcium silicate (Ca_2_O_4_Si) (COD 2,310,675) corresponding with the manufacturer composition description for this material. TCS powder showed peaks of tricalcium silicate (Ca_3_O_5_Si) (COD 1,540,704) and dicalcium silicate (Ca_2_O_4_Si) (COD 1,535,815) as well as a peak corresponding with calcium hydroxide (CaOH_2_) (COD 1,529,752). XRD of pure hydroxyapatite powder exhibited solely the hydroxylapatite phase (Ca_5_HO_13_P_3_) (COD 9,002,213) whilst the HAp-Bi showed additional peaks corresponding with bismuth oxide (Bi_2_O_3_) (COD 9,012,546) addition.

#### Characterization of set materials

**Figure 3:**
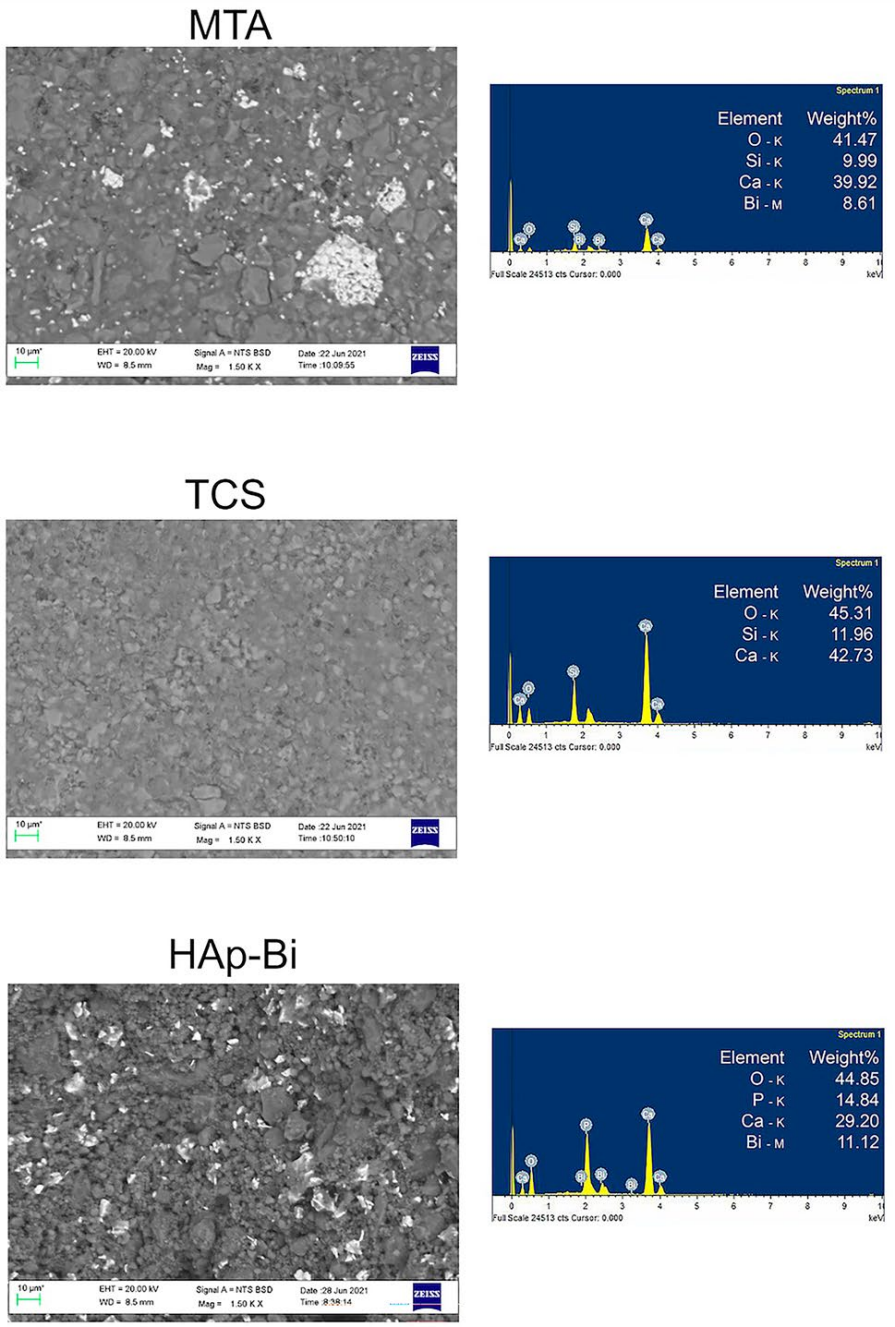

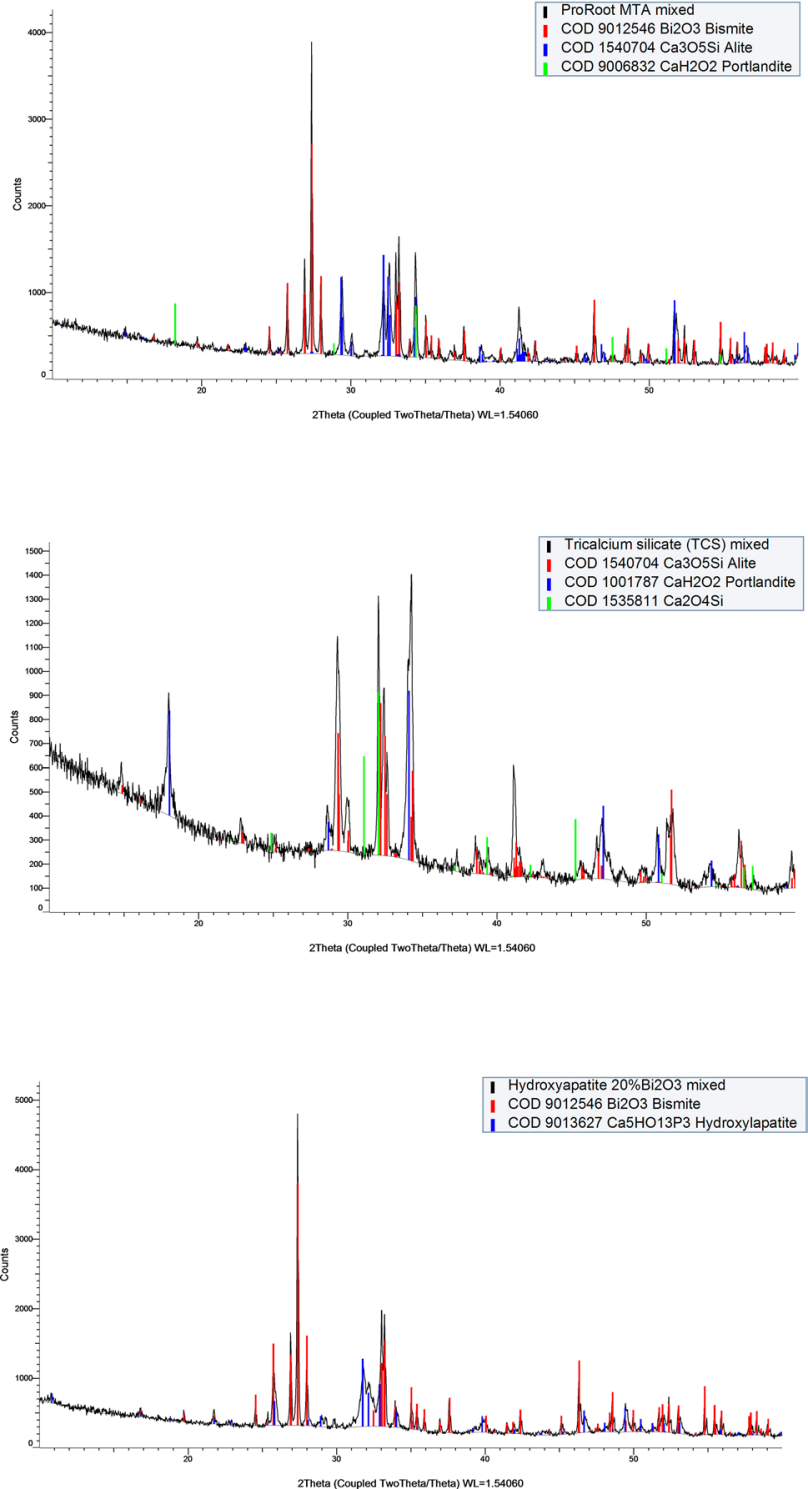
(**a**) SEM/EDS and (**b**) XRD characterization of the material set samples.

SEM/EDS and XRD analysis of the materials after hydration are shown in Fig. 3a,b respectively. MTA was composed of well defined cement particles with some hydration products and bismuth oxide particles interspered in the cement matrix. TCS particles were smaller than the MTA and showed a higher degree of hydration with hydration products filling the spaces between particles. HAp-Bi exhibited irregular sized particles with the presence of bismuth-containing particles interposed in the material matrix. XRD of hydrated MTA exhibited peaks of tricalcium silicate (Ca_3_O_5_Si) (COD 1,540,704), bismuth oxide (Bi_2_O_3_) (COD 9,012,546) and peaks corresponding with the precipitated hydration product calcium hydroxide (COD 9,006,832). XRD of TCS showed peaks of tricalcium silicate (Ca_3_O_5_Si) (COD 1,540,704), dicalcium silicate (Ca_2_O_4_Si) (COD 1,535,811) and calcium hydroxide (COD 1,001,787). The hydrated hydroxyapatite XRD exhibited the hydroxylapatite phase only (Ca_5_HO_13_P_3_) (COD 9,002,213), whilst HAp-Bi XRD revealed a peak for the replacement (Bi_2_O_3_) (COD 9,012,546).

### *Assessment of *in vivo* elemental dispersion and tissue inflammatory response*

#### Animal-to-human age equivalency

The weight assessments indicated an increase in weight for all animals. The equivalence analysis for the initial, 30 and 180 days of age from the animals with human age are represented in Supplemental Table A. The animal-to-human equivalence indicated a correspondent period of three years for the 30-day implantation and approximately nineteen years for the 180-day implantation.

#### Local elemental release

**Figure 4:**
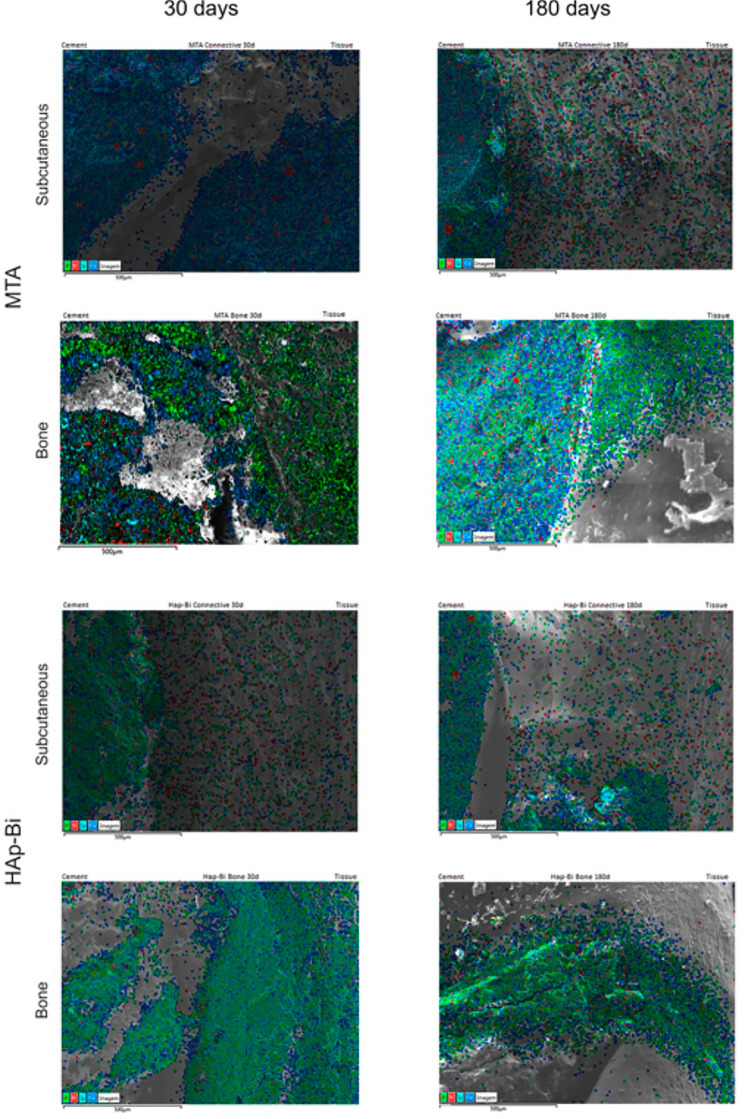

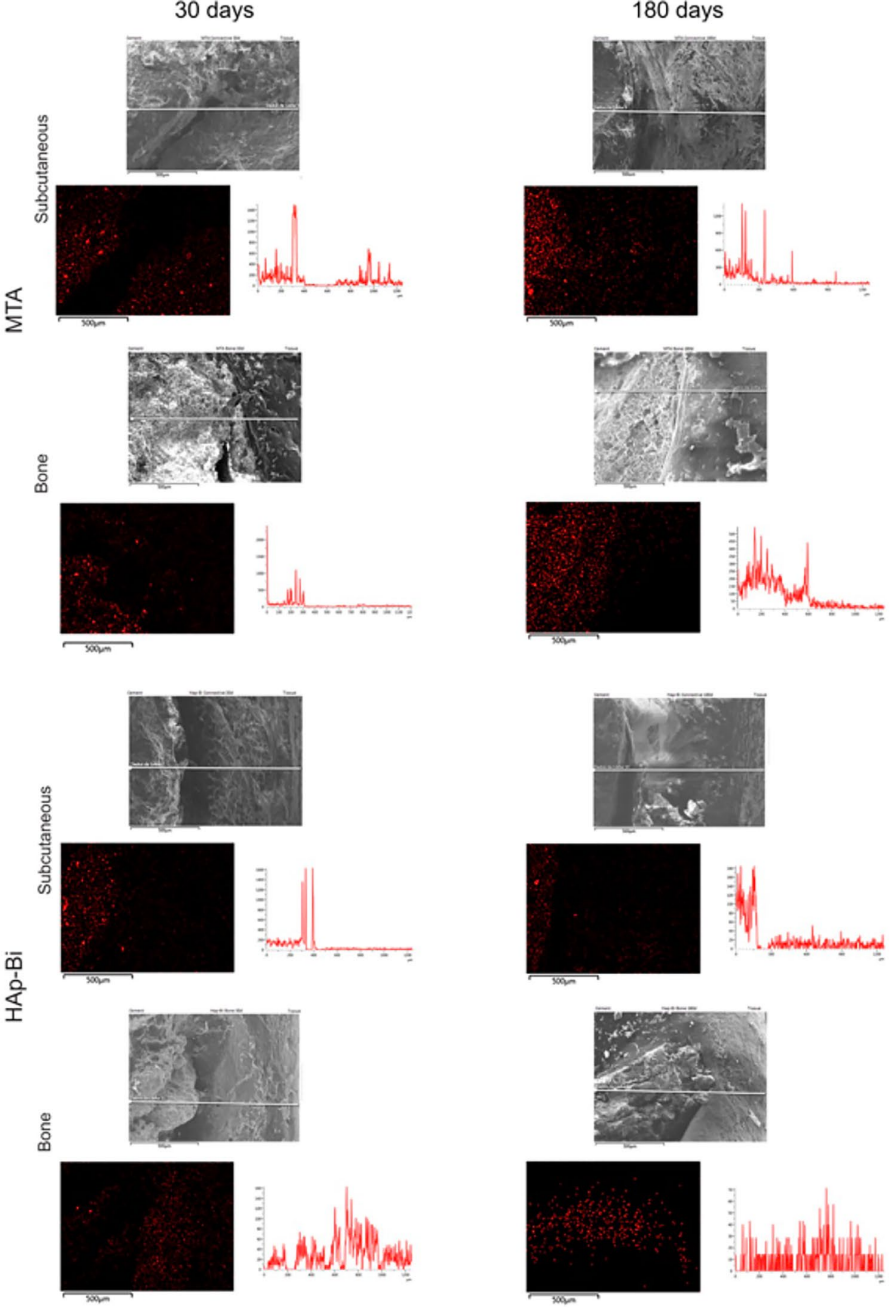

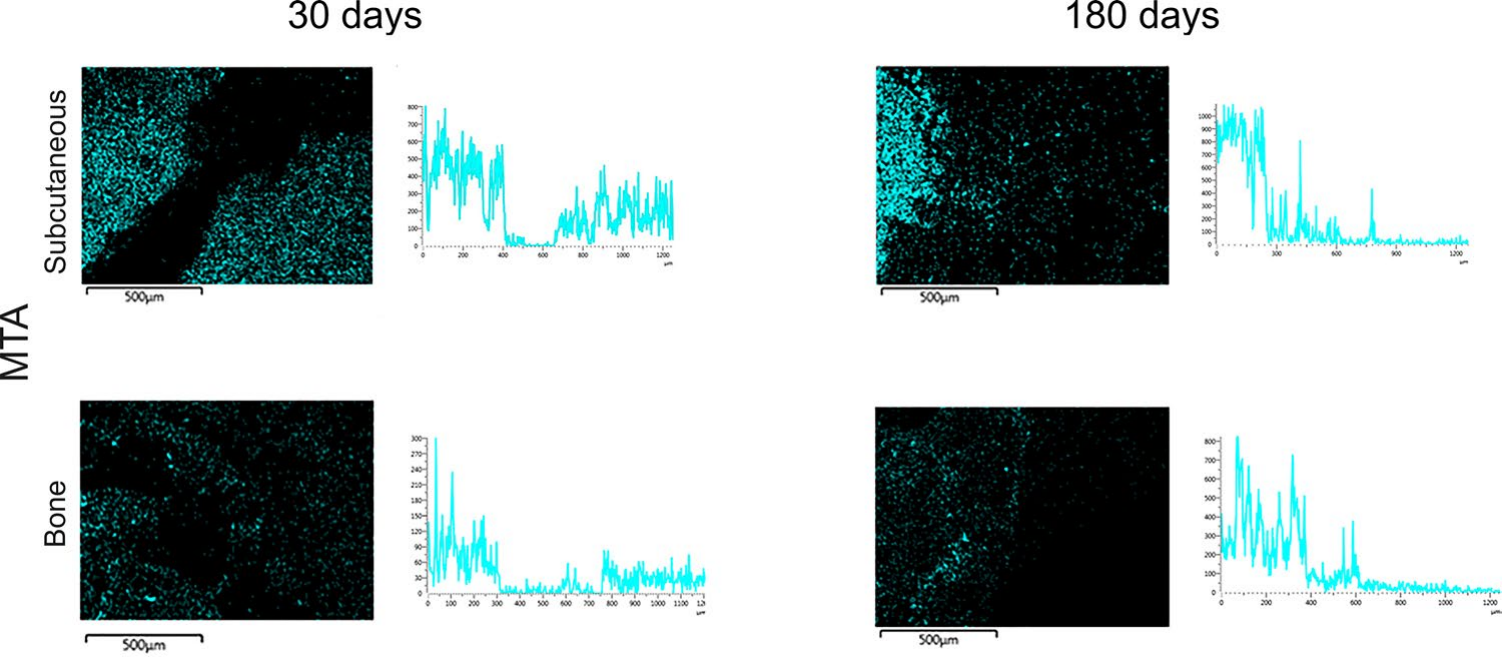
Local element analysis after 30 and 180 days of subcutaneous and bone implantations. (**a**) EDS maps overlapped with SEM microphotographs of bismuth-containing materials and tissue interfaces. Bismuth (red dots) was detected. (**b**) EDS line analysis and bismuth maps indicating the release of this element from the materials into the adjacent tissues. (**c**) MTA silicon maps (light-blue dots) and EDS line graphs.

Bismuth was identified in the subcutaneous tissues surrounding MTA and HAp-Bi implants after 30 days and 180 days (Fig. 4a) with the signal observed along the EDS line (Fig. 4b) and a quantifiable element weight detected for bismuth. SEM micrographs, elemental maps and line EDS analysis of TCS and control samples without implantation are shown in Supplemental: Fig. A for subcutaneous implantation at 30 days, Fig. B for subcutaneous implantation at 180 days, Fig. C for intraosseous implantation at 30 days and Fig. D for intraosseous implantation at 180 days. In the retrieved bone, bismuth was detected only from MTA samples, whereas for HAp-Bi no bismuth was detected. No bismuth was detected regardless of the tissue or the implantation period for either TCS or controls. Silicon peaks were detected in both bone and subcutaneous tissues associated with the MTA samples after 30 and 180 days (Fig. 4c). Silicon was not detected in the HAp-Bi. Minimal detection of silicon was found in bone tissue whilst control samples exhibited silicon in the subcutaneous tissues but not in bone.

**Figure 5:**
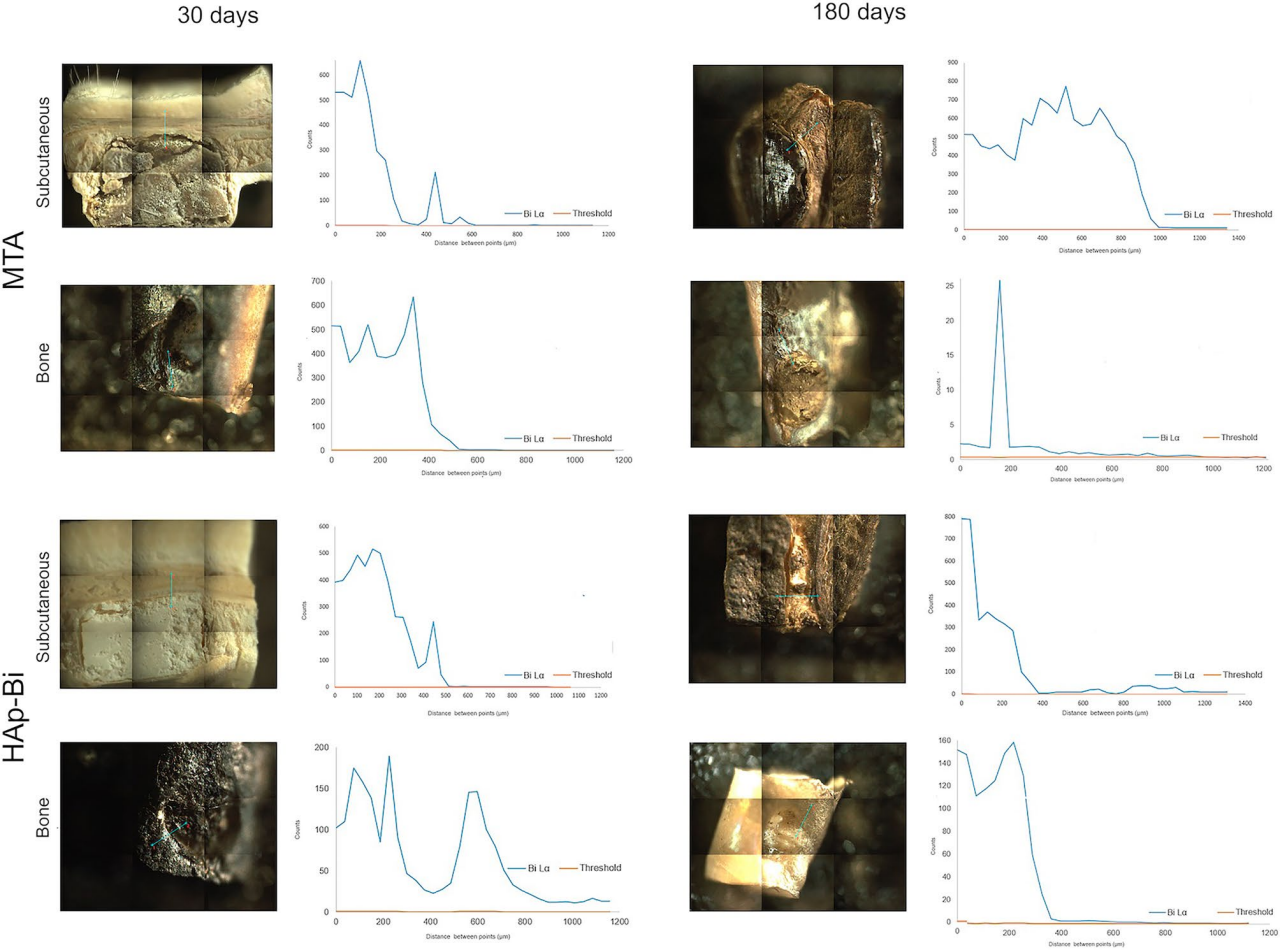
m-XRF analysis of of bismuth-containing materials and tissues interface after 30 and 180 days of subcutaneous and bone implantations. Bismuth was detected migrating from the material into the adjacent tissues.

The m-XRF analysis of subcutaneous tissue and bone samples for both periods are shown in Fig. 5 and Supplemental Figs. E–H. In the MTA in contact with subcutaneous tissues for 30 days, bismuth was detected in both the material and at lower intensity in surrounding tissue, whereas after 180 days a higher intensity of bismuth was found in the tissue indicating further release over time. A similar finding was observed for HAp-Bi. Bismuth was detected in negligible amounts for both the TCS and control groups at all periods. In bone samples, for the MTA and HAp-Bi at both periods, bismuth was identified in the material and at a low intensity in the surrounding tissue with a reduction in the local bismuth over time, as observed in the EDS line analysis graphs and bismuth mappings (Fig. 5). Bismuth was not detected in the TCS and control samples.

The local analysis of mass fractions of bismuth detected using ICP-MS are represented in Table 1a and those of silicon in Table 1b. Bismuth was detected in both tissues surrounding the MTA and HAp-Bi in both evaluated periods. A higher mean mass fraction was observed for HAp-Bi after 30 days in the subcutaneous tissues in comparison with the 180-day period. No statistical differences were found between MTA and HAp-Bi (*p* > 0.05) for bismuth. Silicon was detectable in all groups, with higher mass fraction in the subcutaneous tissue after 30 days for TCS and MTA without statistically significant differences (*p* > 0.05) between groups. For all samples, the silicon mass fractions were lower in bone in comparison with the subcutaneous tissues. Calcium and phosphorus were detected at higher intensity in bone with significant differences in comparison with subcutaneous tissues (*p* < 0.05).

**Table 1:** ICP-MS mass fraction (ng/g) of (a) bismuth and (b) silicon detected in the material-implanted adjacent local tissue (subcutaneous or bone) samples (n = 5). For controls (n = 3) no material implantation occurred.

Bismuth (ng/g)					
	Local				
	Mean	SD	Min	Max	Median
ProRoot MTA					
Subcutaneous 30 days	**928.72** ac	2048.710	1.72	4593.52	10.44
Bone 30 days	**187.87** a	212.716	37.34	553.39	80.27
Subcutaneous 180 days	**475.74** ac	437.133	6.59	1029.82	471.02
Bone 180 days	**213.49** a	342.313	16.73	819.98	56.79
Hydroxyapatite 20% Bi_2_O_3_					
Subcutaneous 30 days	**8249.23** ac	15,695.924	6.54	36,198.13	1293.11
Bone 30 days	**730.05** a	632.636	193.61	1822.01	552.41
Subcutaneous 180 days	**13.79** c	14.104	1.64	38.07	9.29
Bone 180 days	**319.19** ac	647.422	11.97	1476.56	24.15
Tricalcium silicate					
Subcutaneous 30 days	**0.31** b	0.226	− 0.04	0.47	0.45
Bone 30 days	**0.37** b	0.281	0.07	0.80	0.28
Subcutaneous 180 days	**0.59** b	0.304	0.10	0.86	0.63
Bone 180 days	**0.02** d	0.136	− 0.06	0.26	− 0.03
Controls (no material)					
Subcutaneous 30 days	**0.40** bd	0.417	0.12	0.88	0.20
Bone 30 days	**0.03** bd	0.097	− 0.04	0.14	− 0.0002
Subcutaneous 180 days	**0.28** bd	0.570	− 0.05	0.94	− 0.05
Bone 180 days	**0.14** bd	0.238	− 0.05	0.41	0.06

Silicon (ng/g)					
	Local				
	Mean	SD	Min	Max	Median
ProRoot MTA					
Subcutaneous 30 days	**1156.37 ** ad	880.311	540.05	2699.93	805.15
Bone 30 days	**328.32 ** bcgh	125.827	182.16	488.90	277.19
Subcutaneous 180 days	**679.27 ** acg	232.631	445.79	1030.97	641.36
Bone 180 days	**316.82** begh	159.060	218.19	595.47	255.74
Hydroxyapatite 20% Bi_2_O_3_					
Subcutaneous 30 days	**664.65** afg	160.075	502.66	878.42	699.65
Bone 30 days	**287.42** b	34.808	240.76	327.91	280.33
Subcutaneous 180 days	**464.74** g	72.965	366.38	557.10	446.85
Bone 180 days	**16.61** h	15.078	202.53	239.44	217.71
Tricalcium silicate					
Subcutaneous 30 days	**671.10** df	6163.282	693.72	15,112.46	8202.52
Bone 30 days	**292.38** bi	66.254	224.32	367.55	267.74
Subcutaneous 180 days	**539.74** aceg	185.207	380.09	839.58	487.35
Bone 180 days	**235.38** hi	21.026	213.71	265.73	229.67
Controls (no material)					
Subcutaneous 30 days	**611.70** aegj	79.135	531.25	689.46	614.38
Bone 30 days	**222.30** bhj	21.557	201.37	244.43	221.08
Subcutaneous 180 days	**384.17** bcgj	80.012	301.13	460.76	390.61
Bone 180 days	**220.01** bij	29.496	200.66	253.96	205.41

Different lowercase letters represent differences between implantation site and time of analysis.

SD standard deviation.

Significant value are in value bold.

#### Systemic elemental release

The blood and organs mass fractions of bismuth and silicon are shown in Table 2a, b respectively. For the bismuth-containing materials, high levels of this element were identified systemically and accumulated in the kidney, with a higher mass fraction being observed for HAp-Bi implanted in bone for 30 days in comparison with the two other materials (*p* < 0.05). After 180-day implantation, no statistical differences were found between subcutaneous or bone tissue implantation for MTA and HAp-Bi of bismuth accumulation in the kidneys (*p* > 0.05). Bismuth mass fractions were also quantified in blood, liver and brain for both MTA and HAp-Bi groups although in smaller amounts in comparison with the kidney levels (*p* < 0.05). No bismuth could be detected in the water nor wood shavings used for animal welfare and a mass fraction of 0.09 ng/g was detected in the food provided.

**Table 2:** ICP-MS mass fraction (ng/g) of (a) bismuth and (b) silicon detected in whole blood, liver, brain and kidney samples (n = 5) after 30 and 180 days of implantation. For controls (n = 3) no material implantation occurred.

	Bismuth (ng/g)																			
	Blood					Liver					Brain					Kidney				
	Mean	SD	Min	Max	Median	Mean	SD	Min	Max	Median	Mean	SD	Min	Max	Median	Mean	SD	Min	Max	Median
ProRoot MTA																				
Subcutaneous 30 days	**1.14** A a	1.011	− 0.02	2.55	1.37	**5.21** B a	3.870	2.07	11.31	3.43	**0.75** A a	0.531	0.004	1.47	0.70	**334.42** C ad	264.507	116.51	777.75	277.62
Bone 30 days	**BDL**	0.037	− 0.47	− 0.38	− 0.44	**0.99** A b	0.842	0.06	2.35	0.91	**BDL**	0.396	− 0.51	0.52	− 0.29	**279.38** B ad	145.986	111.77	422.94	364.00
Subcutaneous 180 days	**BDL**	0.039	− 0.22	− 0.14	− 0.21	**0.94** A b	0.569	0.44	1.73	0.76	**0.07** B a	0.183	− 0.12	0.31	0.11	**384.31** C ad	116.326	282.74	563.75	342.56
Bone 180 days	**BDL**	0.028	− 0.49	− 0.43	− 0.49	**BDL**	0.203	− 0.28	0.19	− 0.08	**BDL**	0.091	− 0.33	− 0.09	− 0.27	**85.90** A b	41.360	46.15	149.40	66.12
Hydroxyapatite 20% Bi_2_O_3_																				
Subcutaneous 30 days	**0.17** A a	0.019	0.14	0.18	0.18	**2.24** B a	0.761	1.70	3.55	1.93	**0.83** C a	0.600	0.32	1.87	0.70	**538.25** D ac	156.758	308.36	691.71	561.68
Bone 30 days	**BDL**	0.228	− 0.35	0.17	− 0.33	**19.74** A a	23.692	1.99	50.44	3.29	**0.77** B a	0.702	− 0.17	1.54	0.56	**1065.63** C c	587.962	614.09	2054.46	927.03
Subcutaneous 180 days	**0.52** A a	0.544	− 0.09	1.30	0.57	**0.86** A b	0.370	0.36	1.32	0.84	**0.89** A a	0.687	0.06	1.83	1.01	**286.86** B d	74.185	216.86	403.27	291.28
Bone 180 days	**BDL**	0.007	− 0.50	− 0.49	− 0.49	**BDL**	0.133	− 0.33	− 0.01	− 0.08	**BDL**	0.067	− 0.39	− 0.22	− 0.30	**66.22** A b	20.507	37.39	88.20	62.45
Tricalcium silicate																				
Subcutaneous 30 days	**BDL**	0.012	− 0.14	− 0.11	− 0.14	**BDL**	0.017	− 0.11	− 0.07	− 0.10	**BDL**	0.064	− 0.13	0.03	− 0.09	**BDL**	0.036	− 0.12	− 0.04	− 0.10
Bone 30 days	**0.03** A a	0.008	0.03	0.04	0.03	**BDL**	0.308	− 0.51	0.23	− 0.34	**0.17** B a	0.091	0.05	0.30	0.17	**0.29** B e	0.160	0.11	0.42	0.37
Subcutaneous 180 days	**BDL**	0.316	− 0.27	0.48	− 0.24	**BDL**	0.111	− 0.16	0.11	− 0.13	**BDL**	0.041	− 0.15	− 0.05	− 0.13	**BDL**	0.014	− 0.29	− 0.25	− 0.27
Bone 180 days	**BDL**	0.003	− 0.51	− 0.50	− 0.51	**BDL**	0.010	− 0.50	− 0.47	− 0.48	**BDL**	0.011	− 0.51	− 0.48	− 0.50	**BDL**	0.003	− 0.51	− 0.50	− 0.50
Controls (no material)																				
30 days	**BDL**	0.007	− 0.15	− 0.14	− 0.15	**BDL**	0.036	− 0.14	− 0.07	− 0.10	**0.05** A a	0.041	0.02	0.10	0.03	**BDL**	0.008	− 0.13	− 0.12	− 0.12
180 days	**BDL**	0.007	− 0.14	− 0.13	− 0.14	**BDL**	0.003	− 0.14	− 0.13	− 0.13	**BDL**	0.017	− 0.15	− 0.11	− 0.14	**BDL**	0.005	− 0.15	− 0.14	− 0.15

	Silicon (ng/g)																			
	Blood					Liver					Brain					Kidney				
	Mean	SD	Min	Max	Median	Mean	SD	Min	Max	Median	Mean	SD	Min	Max	Median	Mean	SD	Min	Max	Median
ProRoot MTA																				
Subcutaneous 30 days	**2968.21 A a**	229.417	2769.85	3269.33	2845.22	**3031.29** A a	140.828	2790.95	3151.91	3075.53	**2865.46** A a	120.617	2732.680	3047.81	2823.97	**2907.35** A a	132.252	2784.52	3128.75	2863.52
Bone 30 days	**894.52 AB b**	116.537	765.16	1064.06	913.45	**762.58** AB be	189.270	521.61	971.10	764.98	**801.31** A b	48.776	727.06	862.46	801.31	**908.26** B b	48.605	833.42	965.86	919.67
Subcutaneous 180 days	**548.80 AB cd**	200.287	392.61	873.72	476.92	**469.11** AB c	44.298	421.77	529.37	470.82	**425.65** B c	81.636	361.85	564.43	389.55	**480.44** AB c	70.047	392.21	560.12	461.06
Bone 180 days	**840.15** A bc	39.717	793.73	870.35	867.26	**1008.53** B d	56.918	941.73	1096.81	994.39	**848.19** A b	58.945	761.61	913.08	855.95	**892.84** AB b	155.744	781.36	1164.44	839.84
Hydroxyapatite 20% Bi_2_O_3_																				
Subcutaneous 30 days	**2959.88** A a	204.197	2983.51	3237.38	2959.86	**2865.24** A a	106.942	2694.72	2956.60	2879.79	**2858.74** A a	119.685	2656.77	2965.41	2899.49	**2869.41** A ad	119.320	2692.49	3002.95	2853.47
Bone 30 days	**912.03** A b	109.610	826.60	1100.95	879.95	**893.18** A bd	96.988	762.08	1009.39	873.37	**839.49** A b	68.343	764.25	919.14	815.26	**832.38** A b	103.806	735.65	990.89	806.28
Subcutaneous 180 days	**491.63** A d	55.268	439.87	585.74	477.70	**485.83** A c	96.196	376.62	624.92	487.38	**514.41** A cd	59.769	455.67	588.26	507.60	**444.48** A c	75.328	354.47	528.00	481.91
Bone 180 days	**1046.69** A b	289.202	765.57	1531.57	977.84	**926.38** A bd	77.925	812.84	1022.17	932.58	**851.45** A b	68.216	750.02	931.18	868.31	**941.12** A b	61.780	871.18	1007.19	932.77
Tricalcium silicate																				
Subcutaneous 30 days	**2893.54** A a	252.072	2586.67	3254.20	2858.28	**2800.29** A a	72.242	2692.87	2887.06	2823.02	**2766.34** A a	167.124	2602.67	3026.57	2730.64	**2799.40** A ad	93.233	2700.06	2917.26	2791.06
Bone 30 days	**544.50** A d	183.009	357.30	847.20	523.91	**500.35** A c	117.379	319.30	619.41	507.93	**531.61** A cd	94.908	427.06	665.28	505.36	**475.12** A c	114.850	349.85	662.41	446.83
Subcutaneous 180 days	**2852.94** B a	189.287	2680.45	3148.26	2809.35	**659.90** A e	98.600	540.60	807.03	634.31	**552.23** AC d	56.049	467.13	601.10	579.71	**486.84** C c	43.716	431.38	552.81	480.96
Bone 180 days	**854.43** A b	73.680	734.41	919.42	886.05	**885.29** A bd	126.745	797.25	1092.14	808.72	**811.76** A b	106.064	691.92	976.87	805.45	**960.01** A b	78.763	877.93	1083.56	950.65
Controls (no material)																				
30 days	**2930.69** A a	733.459	2461.59	3775.92	2554.58	**2806.55** A a	88.989	2716.34	2894.27	2809.05	**2864.18** A a	294.922	2622.17	3192.67	2777.68	**2749.25** A ad	73.273	2664.64	2791.94	2791.17
180 days	**2788.18** A a	170.924	2610.14	2950.97	2803.44	**2787.92** Aa	137.953	2628.80	2873.84	2861.13	**2770.71** A a	96.281	2681.75	2872.94	2757.44	**2647.31** A d	83.226	2552.04	2705.91	2683.97

Different lowercase letters represent differences between implantation site and time of analysis. Uppercase letters represent differences between local analysis, blood and organs for each group.

SD standard deviation.

BDL below detection level.

Significant value are in value bold.

**Table 3:**
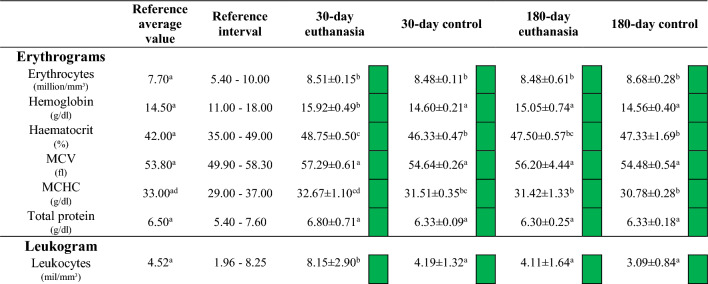
Representation of mean values of the erythrogram analysis, leukograms and biochemical analyses of the hepatic and renal indicators of the euthanized groups in 30 and 180 days compared with respective controls and reference values used^66,67^. Similar letters indicate similarity between the groups.

^2^Colors indicated after the average values for each criterion follow the legend:

Average value within the minimum / maximum reference range.

Average value below the minimum value of the reference range.

Average value above the maximum value of the reference range.

Systemic levels of silicon were comparable with controls after 30 days of subcutaneous implantation for all tested materials (*p* > 0.05). In addition, silicon levels significantly reduced by at least threefold after all bone implantations at both time points and after 180-day subcutaneous implantations. When TCS was implanted subcutaneously, no differences in blood silicon levels could be observed between the time points (*p* > 0.05) but the levels of silicon found in all organs were reduced threefold in comparison with controls, as had been observed for all the bismuth-containing samples.

#### Blood analysis

Analysis of the erythrograms, leukograms and biochemical assays of liver and kidney function obtained at both time periods are shown with the controls alongside previously reported reference values in Table 3. The erythrograms showed similar parameters for all test animals and within the reference values. In the leukogram, the lymphocytes for the 30-day animals were comparable with the control (*p* > 0.05) but significantly higher than the reference values (*p* < 0.05). The hepatic and renal biochemical values for all animals were similar to the reference values at both time points, apart from plasma bilirubin in the 30-day control animals, however gamma-glutamyl transpeptidase was not detected in any samples.

#### Histological assessments

**Figure 6:**
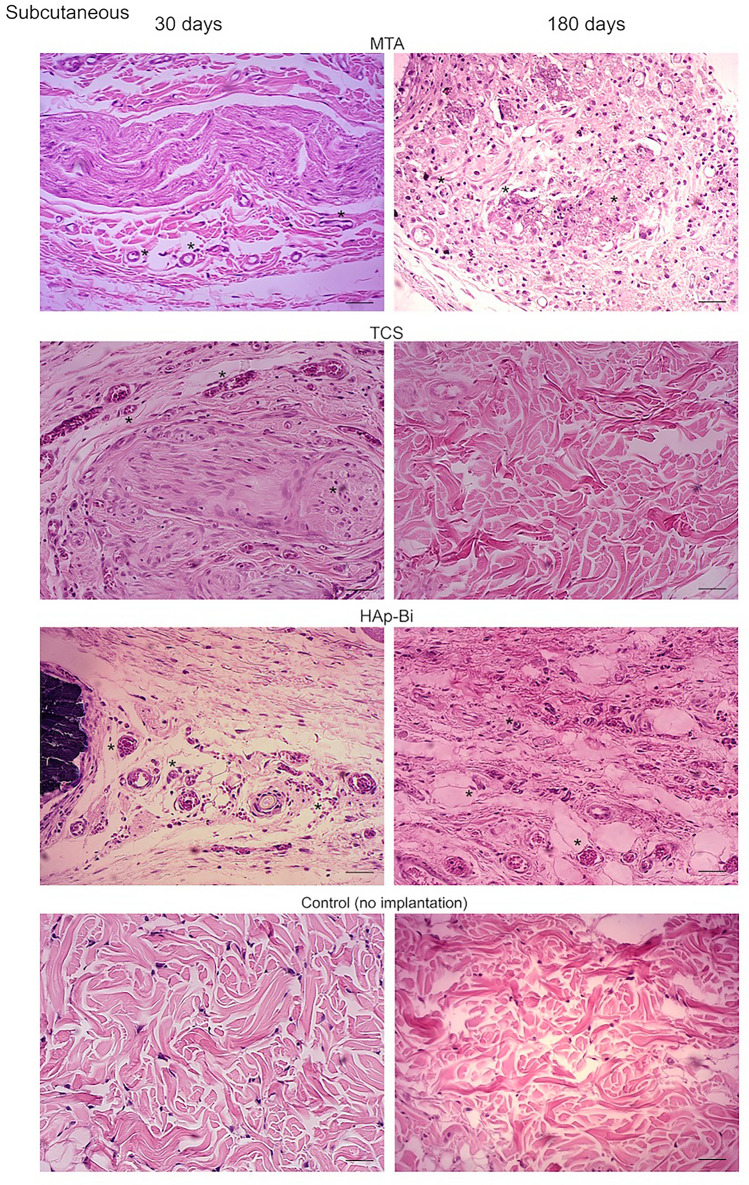

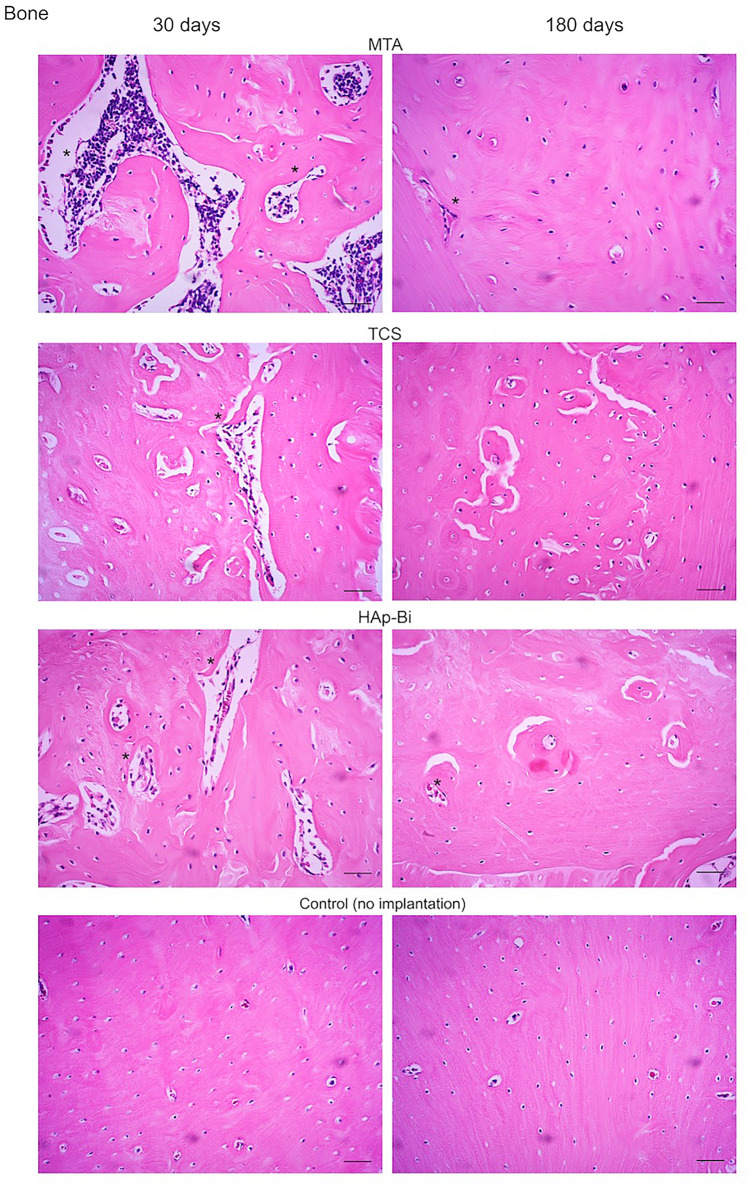
(**a**) Histological sections representative of subcutaneous tissue after 30- and 180-day implantation. (**b**) Histological sections representative of bone after 30- and 180-day implantation. The (*) symbol indicates the tissue alterations after implantation. Controls without implantation for both tissues do not show signs of inflammation. Scale bars represents 20 µm.

**Figure 7:**
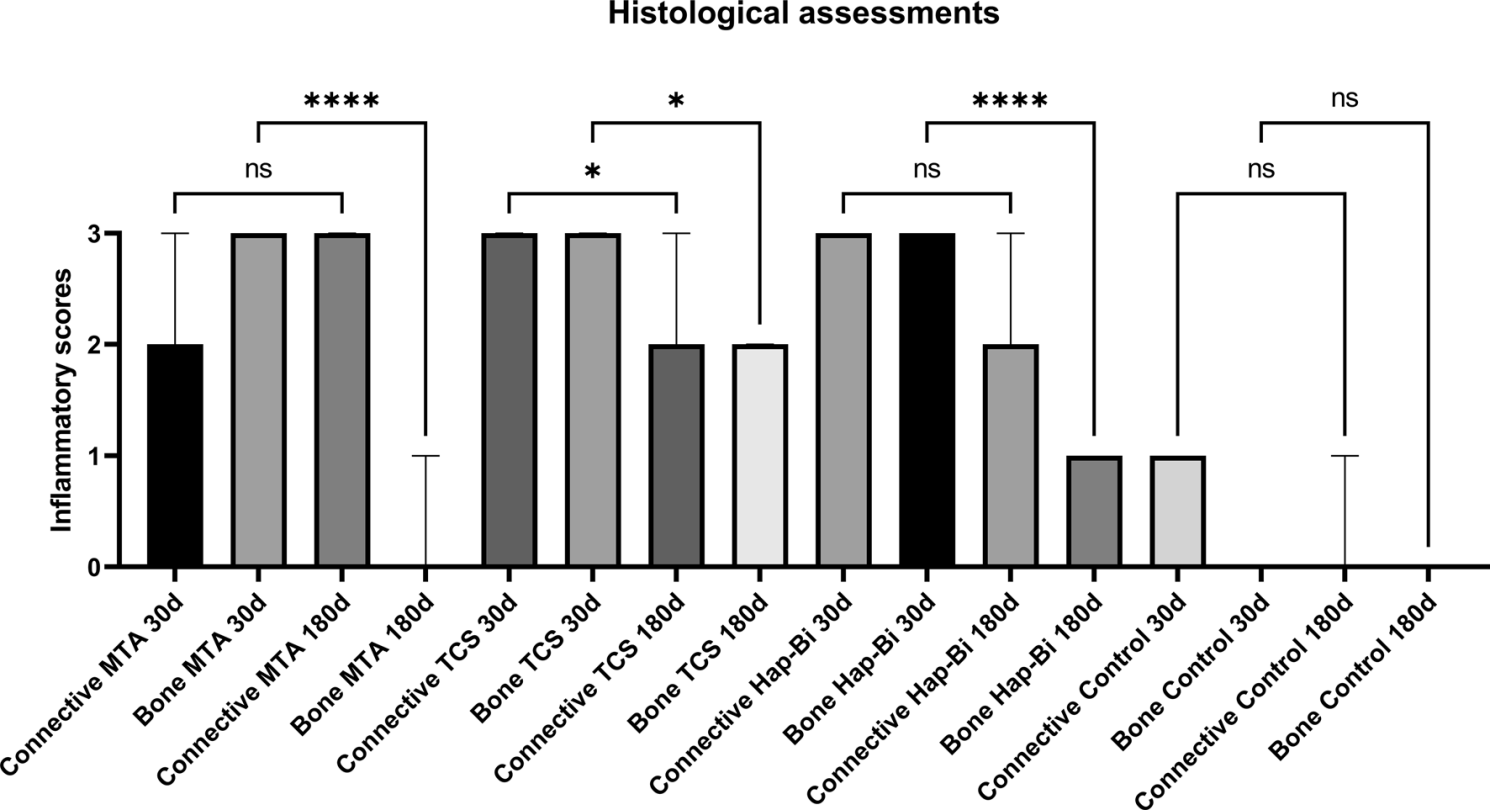
Histological analysis of inflammatory scores for connective and bone after implantation and controls. The (*) symbol represents differences between groups and ‘ns’ stands for no significance.

Representative sections of the local tissues after 30 and 180 days of material implantation are shown in Fig. 6a,b and inflammatory scores related to these sections are presented in Fig. 7. Analysis of subcutaneous tissues after 30-days implantation for all materials exhibited a moderate to intense inflammatory infiltrate, where macrophages and multinucleated giant cells could be observed. After 180 days, the inflammatory infiltrate in MTA and HAp-Bi samples had a chronic appearance. A significant reduction in the inflammatory infiltrate was observed (*p* < 0.05) in the TCS group. After 30 days implantation, all materials in contact with bone appeared to have induced a moderate inflammatory response with the presence of macrophages, lymphocytes and polymorphonuclear cells. After 180 days, for all materials examined the implants into bone showed most indication of repair and a significant reduction in inflammatory scores from 30 to 180 days (*p* < 0.05).

### Material characterization after tissue contact

**Figure 8:**
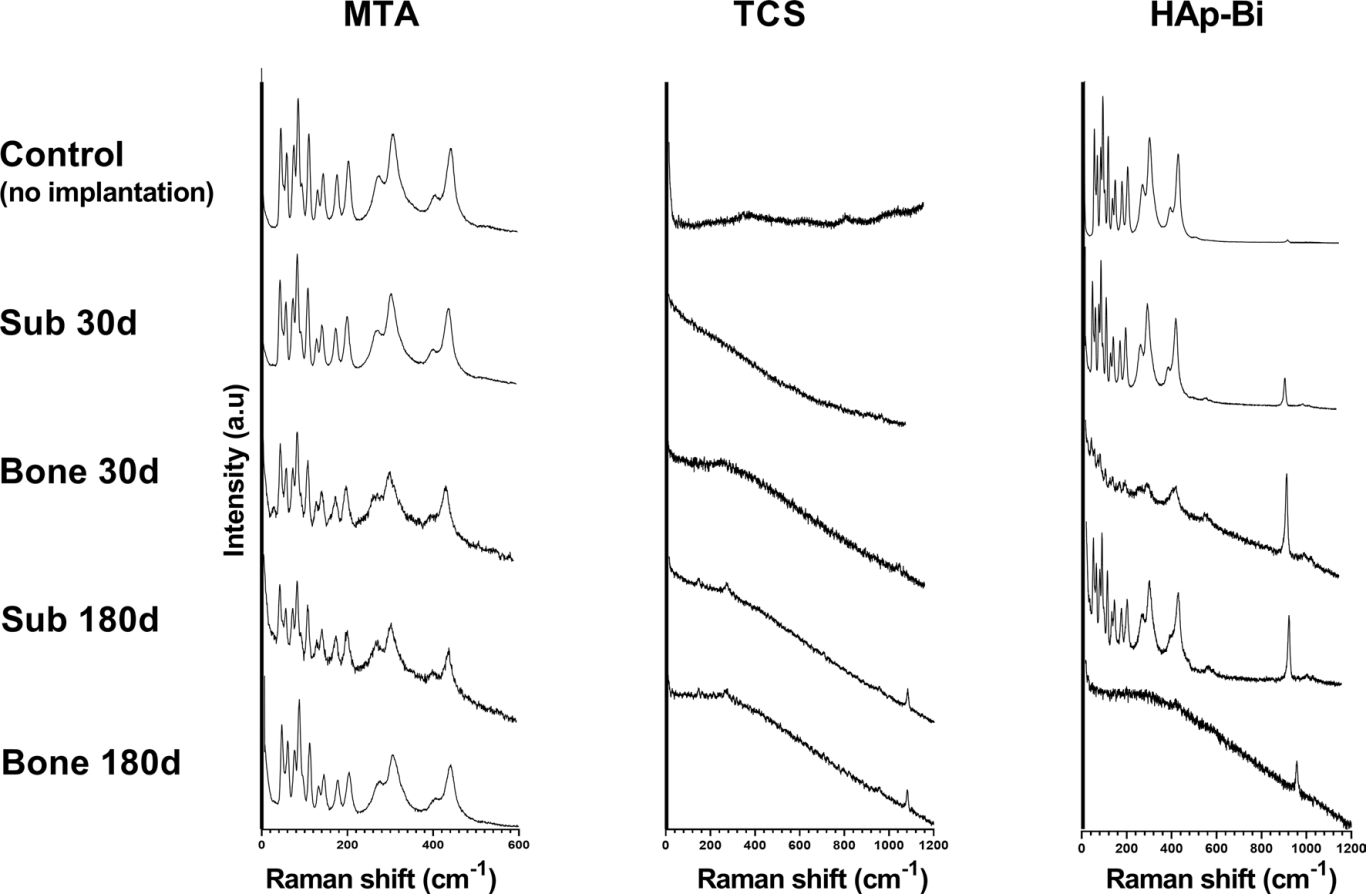
Raman analysis of the implanted sample materials for both implantation periods and tissues in comparison with non-implanted samples.

Raman spectroscopy spectra of the materials after implantation and the non-implanted control samples are shown in Fig. 8. After implantation in subcutaneous tissues, bismuth oxide (Bi_2_O_3_) peaks (103–211, 315 and 412–446 cm^−1^) after 30 and 180 days were still evident for both MTA and HAp-Bi. In addition, characteristic MTA peaks after implantation were comparable with non-implanted control material. For TCS, a carbonation (CaCO_3_) peak was found at 1100 cm^−1^ after 180 days. The HAp-Bi samples exhibited an additional peak corresponding with phosphate (PO_4_) at 958 cm^−1^ after tissue contact; however, this peak was not observed in the corresponding non-implanted control material. Bone implantation of HAp-Bi samples resulted in intense peak alteration between 0 and 500 cm^−1^ in comparison with the non-implanted samples; whilst in contrast, the subcutaneous implantation showed a similar pattern when compared with the non-implanted samples in this range.

## Discussion

The in vivo local and systemic presence of bismuth from tricalcium silicate-based materials after implantation in subcutaneous connective tissue and bone have not previously been investigated. The present study tested the hypothesis that the release of bismuth was dependent on silicon, hence the selection of material compositions used. Bismuth release appeared to occur independently of the material base. Bismuth was detected in local tissues and within the kidneys, even in samples not containing silicon, thus, the null hypothesis was rejected as it was clear that the leaching of silicon and bismuth was not related.

The analysis in the present study was performed in vivo using an animal model, specifically Wistar rats and comparisons between a short- and long-term experimental periods were planned. The sample size determination observed a balance between the 3Rs in animal research (replacement, reduction and refinement)^69^ and a minimal necessary power test^70^ to validate the data obtained by the proposed methods. Selection of male rats was justified based on reported lower hormonal variation when compared with females^71^ since it was not the intention to analyse the accumulation of elements in reproductive organs. Additionally, 12 week-old animals were used corresponding with young humans (9 years-old) whilst the 30-day implantation corresponded with 3 years in humans and the 180-days implantation almost 19 years^17^. These two experimental periods representing a short- and long-term analysis served to establish a potential correspondence with human age.

A number of techniques were employed to assess elemental release which assists with providing a complete picture of material interactions. This is specifically important when evaluating materials based on Portland cement where the substrate interacts with the environment it is placed in, due to the alkalinity and relase of calcium, hydroxyl and silicon from the material into the environment. The interactions of MTA with blood and tissue fluids have been extensively investigated using different methods. The bioactivity potential has been reported whereby calcium and phosphorus interacted forming amorphous apatite crystals on the material surface^72–74^. On the other hand, subcutaneous implantation of MTA was shown to produce surface changes with the deposition of calcium carbonate on the material surface^28^. This latter study was further confirmed by a similar finding investigating material retrieved from a failed apical surgery case^75^. In the animal study^28^, besides calcium carbonate formation, bismuth was shown to be released from subcutaneous tissues into the skin and animal hair. It could be debated that this release and accumulation was artefactual produced by processing sections for histology. This has been shown in a recent study investigating the titanium released from dental implants after immediate loading where the titanium present in the tissues was caused by processing^76^. This is unlikely to be the case in the current study as multiple methods have been used whereby specimens were processed differently and the release was still observed. Furthermore, elements present in peripheral organs could not be caused by contaminaton during processing.

All methods used in the present study confirmed local release of bismuth into both subcutaneous tissues and bone, regardless of the period of implantation. Release of components from ProRoot MTA into dentine^27^ and rat subcutaneous connective tissue^28^ has been previously reported but the present study utilized further methods to fully characterise the release of the bismuth. The material-tissue interface analysis used both line EDS and m-XRF to assess elemental movement from the material to the tissues. Surrounding tissues around the material implant were also acid digested and analysed using ICP-MS which enabled a multi-element chemical analysis^77^, including bismuth. Histological assessments indicated an inflammatory reaction to all implanted materials in the 30-day analysis for both implantation tissues. The inflammatory reaction would cause increased blood flow to the local tissues and therefore potentially increased elemental release systemically. After 180-days, signs of tissue repair could be observed, but the scar tissue was not histologically comparable with the control samples; thus, a level of persistent chronic inflammation was still present even after the long-term analysis.

In the present study, intracardiac-blood and organ retrieval followed by acid digestion allowed quantification and comparison using ICP-MS. For ProRoot MTA, the bismuth mass fraction in blood was detectable only when in contact with subcutaneous tissue for 30 days whilst in all other blood analyses it was not detected. It is debatable whether blood can be representative of the chemical body burden, especially for long-term exposure to non-essential elements, including bismuth^78^. Additionally, the laboratory blood analyses in the present study showed little variation in comparison with reference values and controls, which suggested the systemic accumulation of bismuth might not be reliably measured using blood. However, bismuth accumulation in the kidneys (66- to 1065-fold higher than controls), even after 180 days, indicated how circulating bismuth was markedly deposited in the kidneys. Kidney accumulation of bismuth has been previously reported^65,79^ and can be associated with the strong binding effect of this metal to the kidney metallothionein—a cysteine-rich and low molecular weight protein present in the kidney—after bismuth exposure^80,81^. Kidney impairment followed by heavy metal exposure has been reported to depend on the metal source and amount in a time-dependent manner^82^. The effects of bismuth accumulation in kidney have not been fully elucidated to date. Analysis of animal food and bedding showed low values for bismuth and indicated that accumulated mass fractions for this element were from the material and not from any external environmental source. This was corroborated by Raman analysis of the samples after implantation that showed signs of alterations for all the implanted materials, regardless of implantation site or the composition in comparison with non-implanted controls.

The silicon systemic mass fractions in the present study were influenced by implantation site and the presence of bismuth in the material. Silicon is a metalloid present systemically and is associated with tissue health; however, its specific biochemical process, physiological function or chemical interactions is largely unknown^83,84^. In the present study, a threefold reduction in silicon levels was observed for all systemic samples, except for TCS—not containing bismuth—implanted subcutaneously where blood silicon levels remained comparable with controls after 180 days. For all bone-implanted samples a reduction in silicon levels was also observed, including the blood level, indicating an influence from implantation site on its systemic levels. Inclusion of HAp-Bi in the present study intended to serve as a bismuth-containing material without silicon in its composition and a similar downregulation of silicon levels occurred, indicating the influence of bismuth on silicon levels. Bismuth release after implantation regardless of the material composition may be an indication of the instability of bismuth compounds.

The present study described in vivo the lack of chemical stability represented by local release of bismuth into surrounding tissues regardless of the implantation site as well as the accumulation of bismuth in kidneys following ProRoot MTA implantation. The ICP-MS analysis exhibited a higher variability—indicated by the calculated standard deviation—in the local analysis in comparison with the systemic one; which was an indication of the variable degree of separation of the sample from the tissue before acidic digestion needed for ICP-MS analysis. No studies have considered the potential side-effects of this long-term bismuth kidney accumulation following use of this dental material. Previous studies suggested banning bismuth oxide and its replacement with alternative radiopacifiers. This was done to prevent the discolouration of adjacent tissues by bismuth-containing materials^25,27,85,86^. The lack of stability of this compound was extensively described in different scenarios^31^. In pharmaceutical research, other substances such as bismuth salicylate salts were also associated with biological tissue discolouration of the tongue^87^ and darkening of the faeces^88,89^ after its oral administration. Additionally, an animal study reported darkening of the kidney due to a long-term bismuth salt oral administration for eight years^90^.

## Conclusion

Bismuth was released from materials into adjacent tissues, was detected in the blood and also accumulated in the peripheral organs. This release was independent of the presence of silicon and occurred at both time periods. The use of bismuth in dental materials should be restricted.

## Supplementary Information

Supplementary Information.

## Data Availability

All obtained data regarding this work is presented in this manuscript. The Supplementary material associated with this manuscript can be found at the file provided.
