# Supplementary material for: Bismuth release from endodontic materials: in vivo analysis using Wistar rats
Source: Sci Rep. 2023 Jun 15;13:9738. doi: 10.1038/s41598-023-36690-4 (PMC10272127; doi:10.1038/s41598-023-36690-4)

Bismuth migration from endodontic materials – *in vivo* analysis

Supplemental material to the main manuscript:

Supplemental Table A - Correlation between rat/human age that comprises the analysed periods of 30 and 180 days

Experimental period	Animals' age (weeks)	Animals' age (months)	Animals' age (years)	Correspondent age in humans (years)	Representative implant time in humans (years)
Initial	6	1,50	0,125	4,50	-
Surgical procedures	12	3,00	0,250	9,00	0,00
30-day Euthanasia	16	4,00	0,333	12,00	3,00
180-day Euthanasia	37	9,25	0,770	27,75	18,75

Supplemental Figure A - SEM micrographs, elemental maps, and line EDS analysis for TCS and control samples (without implantation) for subcutaneous implantation at 30 days.

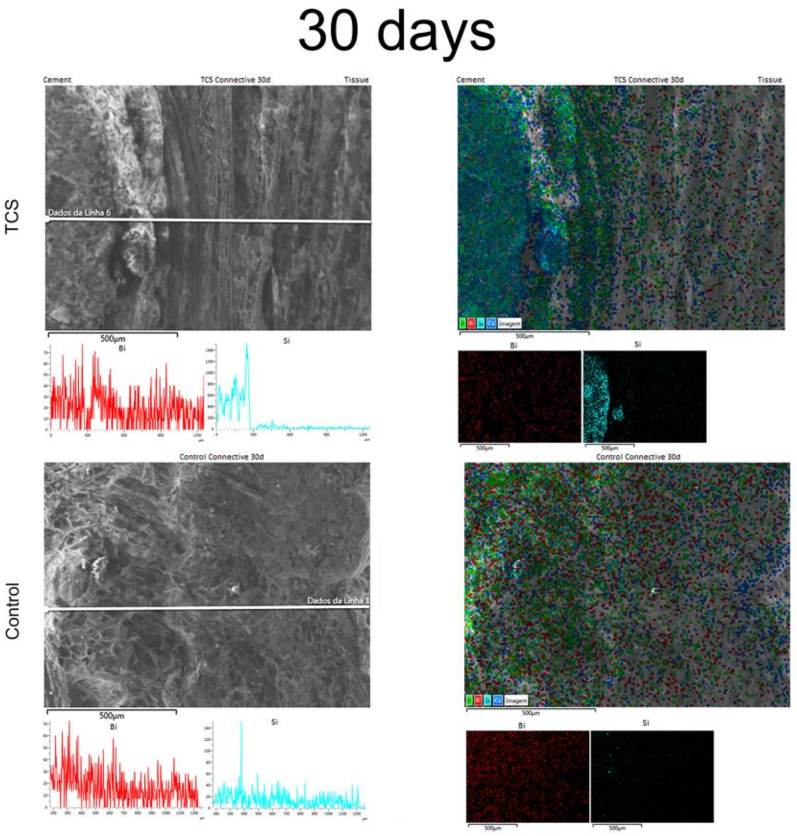

Supplemental Figure B - SEM micrographs, elemental maps, and line EDS analysis for TCS and control samples (without implantation) for subcutaneous implantation at 180 days.

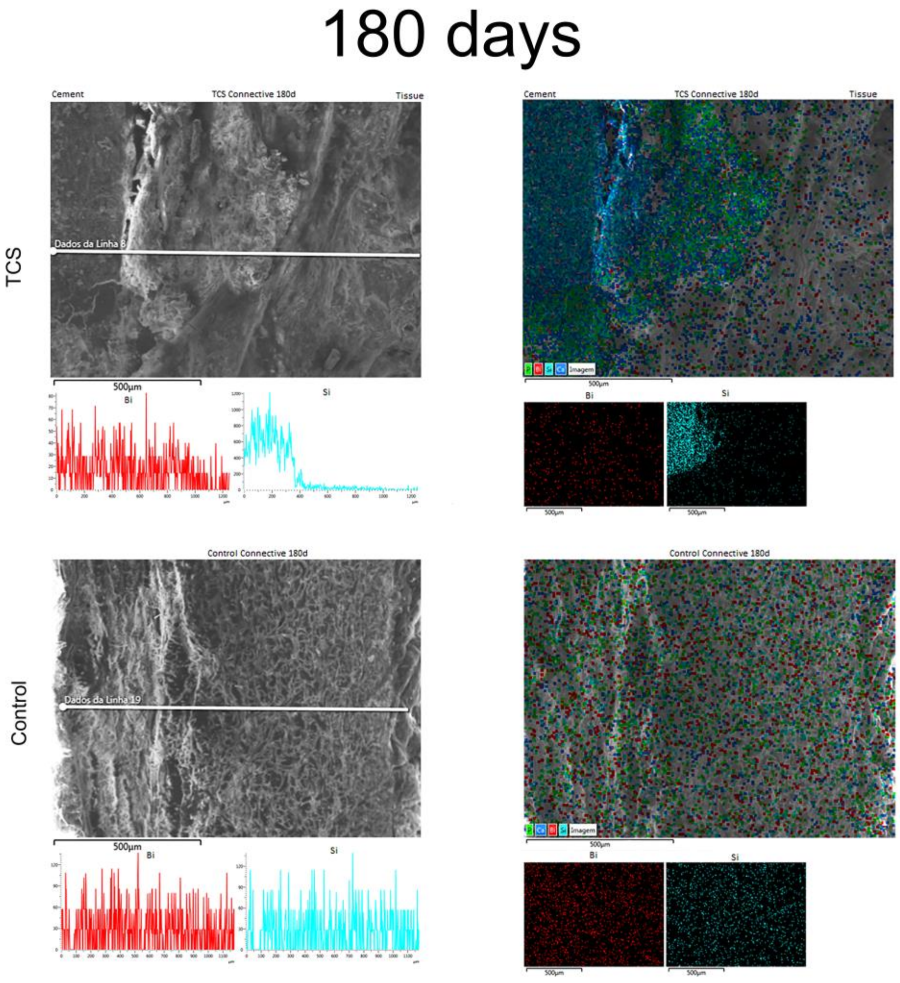

Supplemental Figure C - SEM micrographs, elemental maps, and line EDS analysis for TCS and control samples (without implantation) for bone implantation at 30 days.

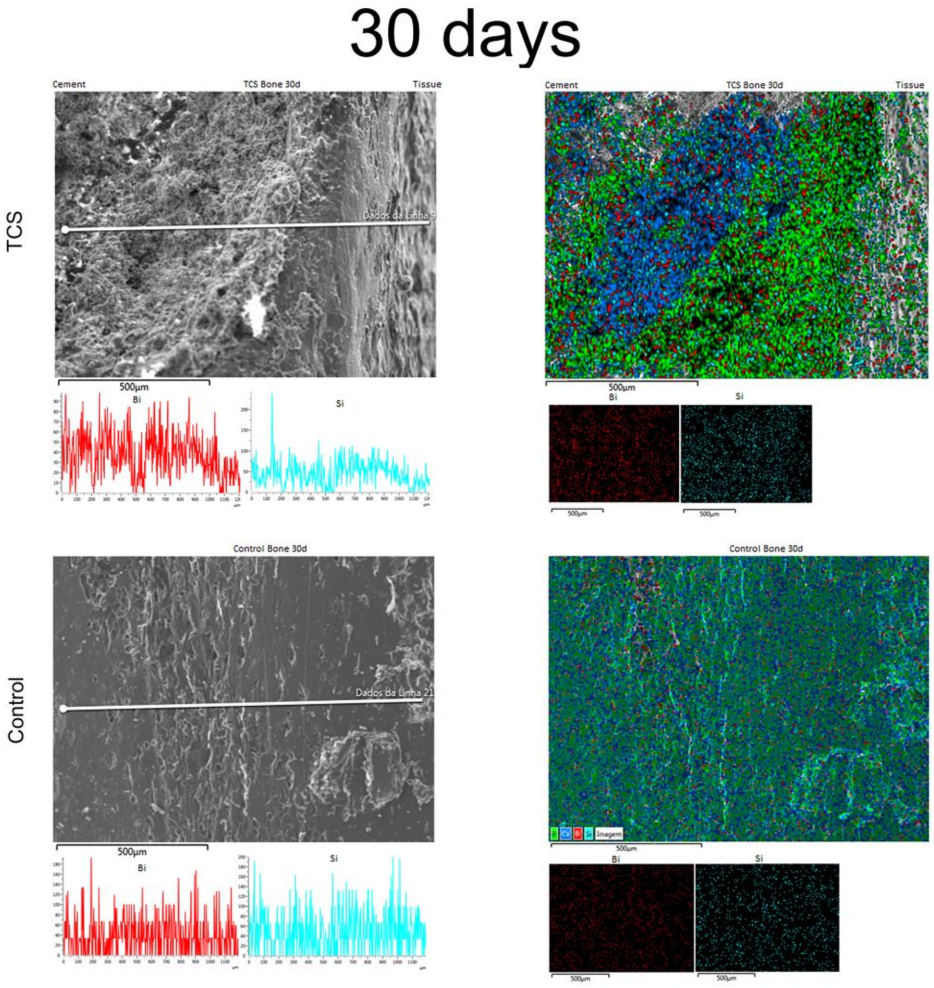

Supplemental Figure D - SEM micrographs, elemental maps, and line EDS analysis for TCS and control samples (without implantation) for bone implantation at 180 days.

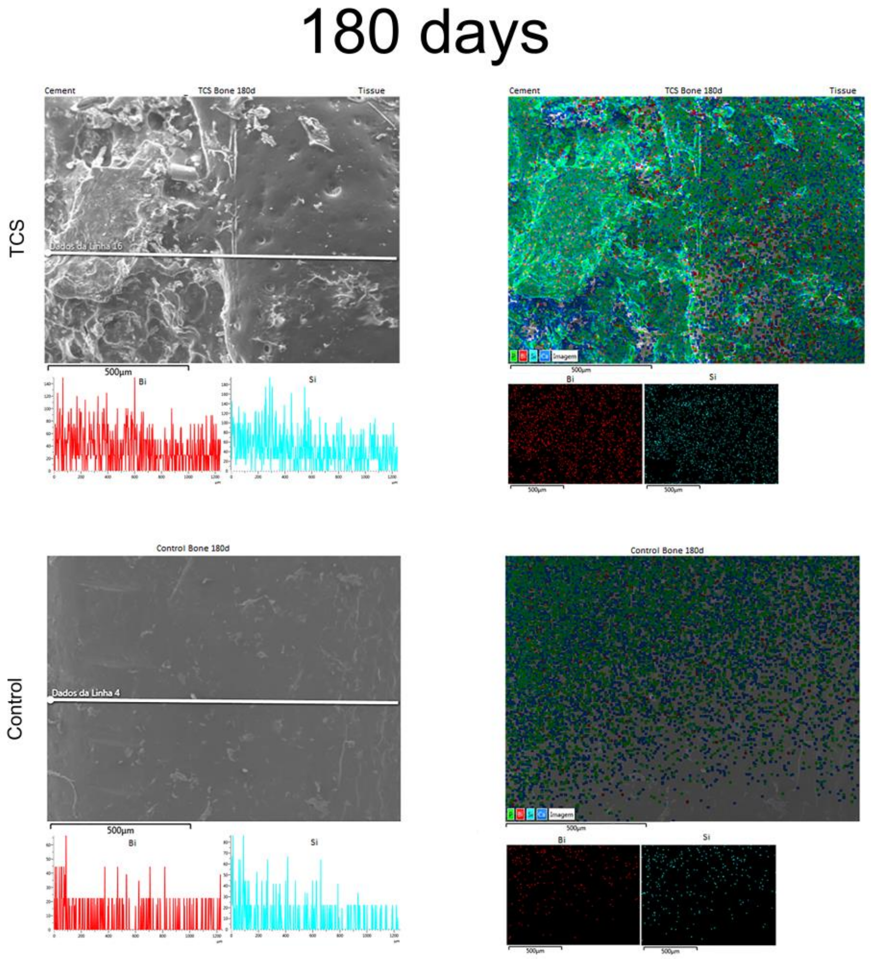

Supplemental Figure E - m-XRF analysis for TCS and control samples (without implantation) for subcutaneous implantation at 30 days.

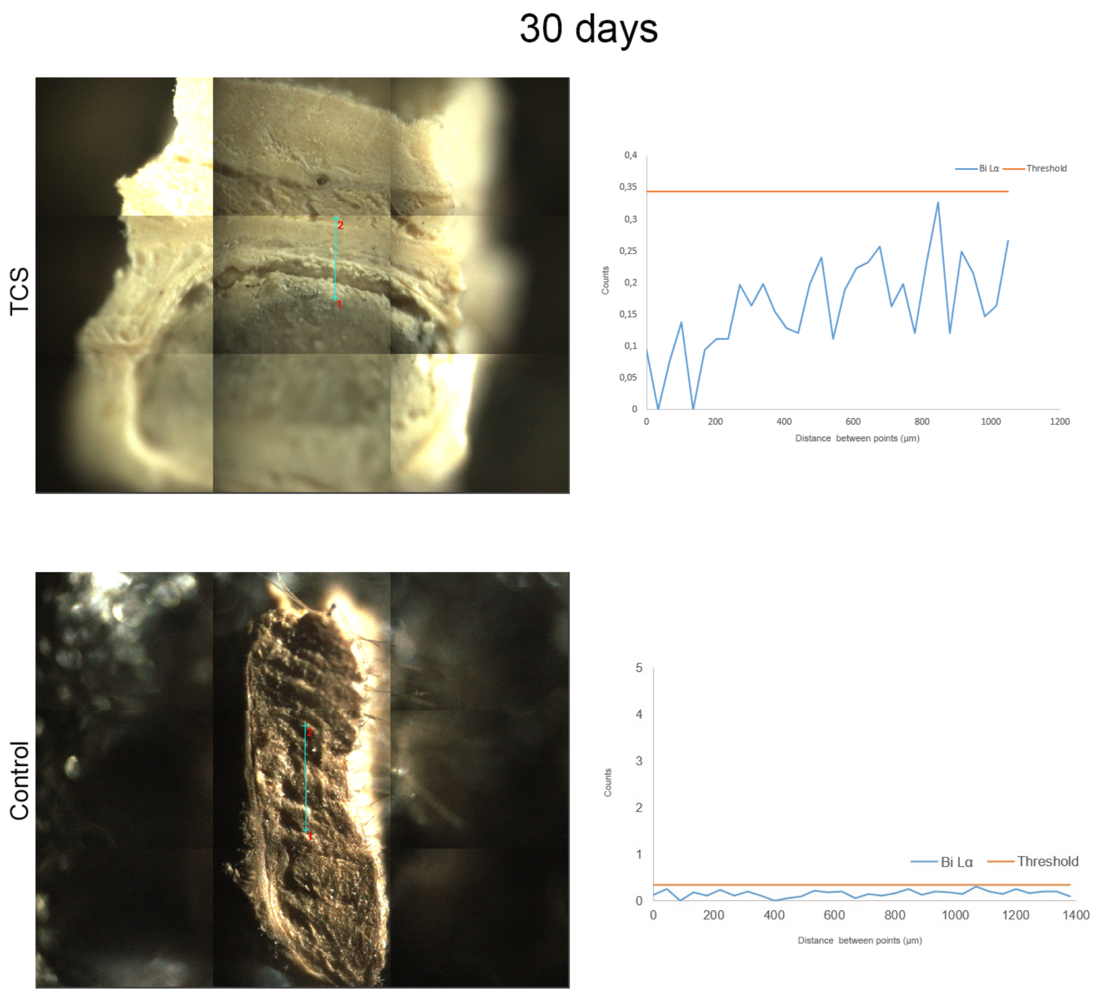

Supplemental Figure F - m-XRF analysis for TCS and control samples (without implantation) for subcutaneous implantation at 180 days.

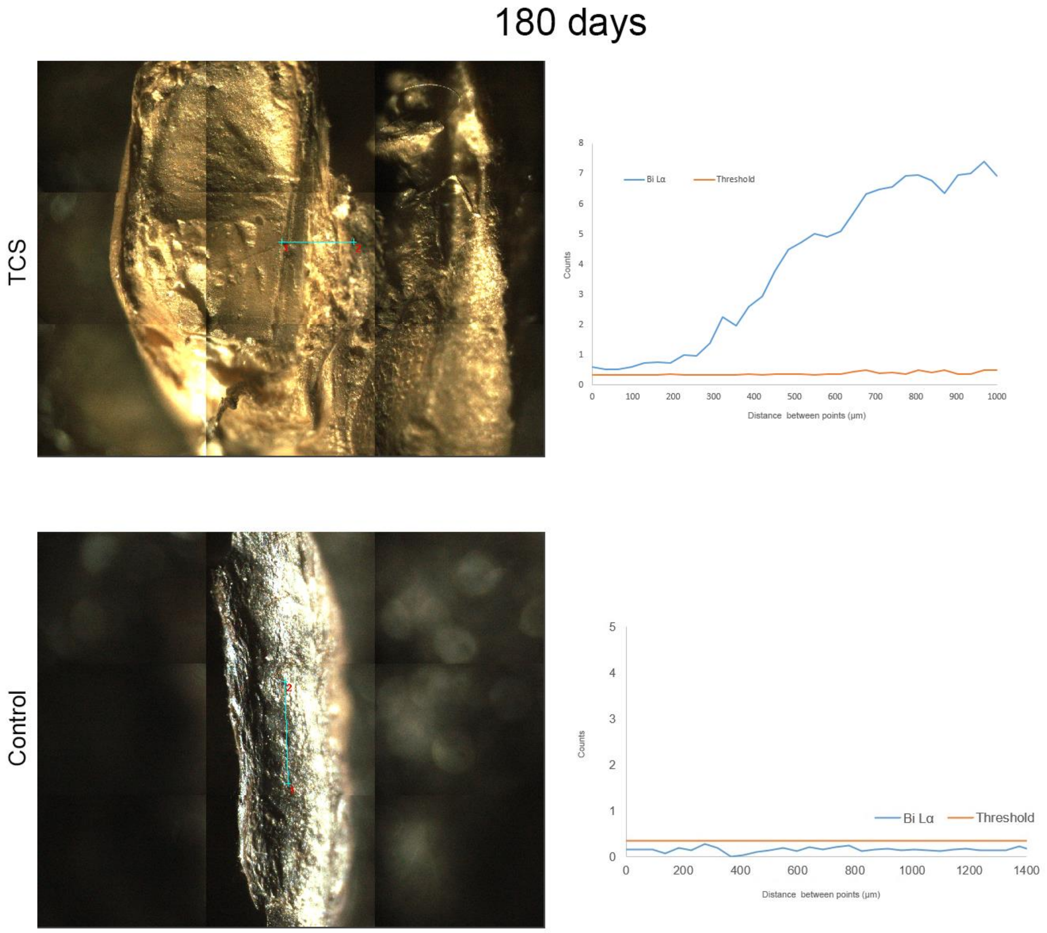

Supplemental Figure G - m-XRF analysis for TCS and control samples (without implantation) for bone implantation at 30 days.

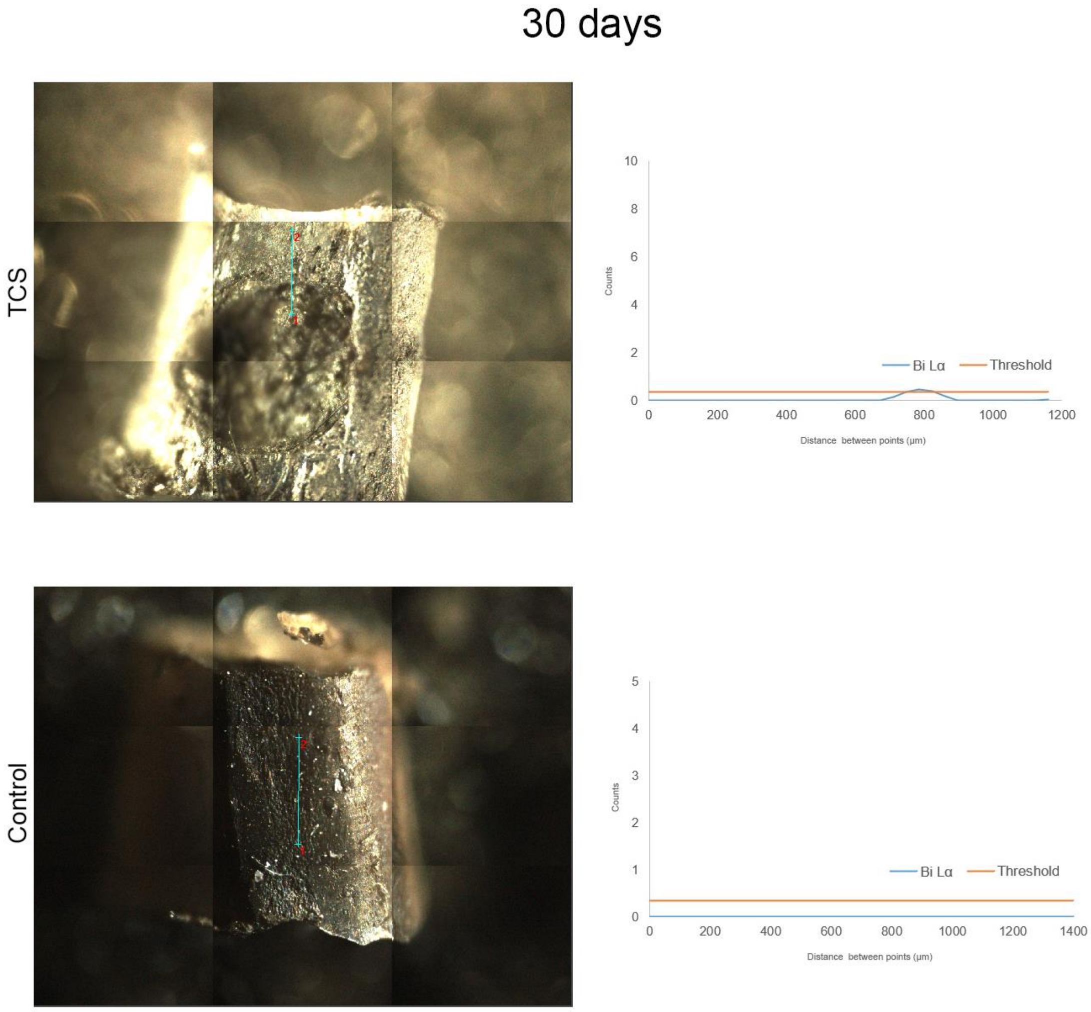

Supplemental Figure H - m-XRF analysis for TCS and control samples (without implantation) for bone implantation at 180 days.

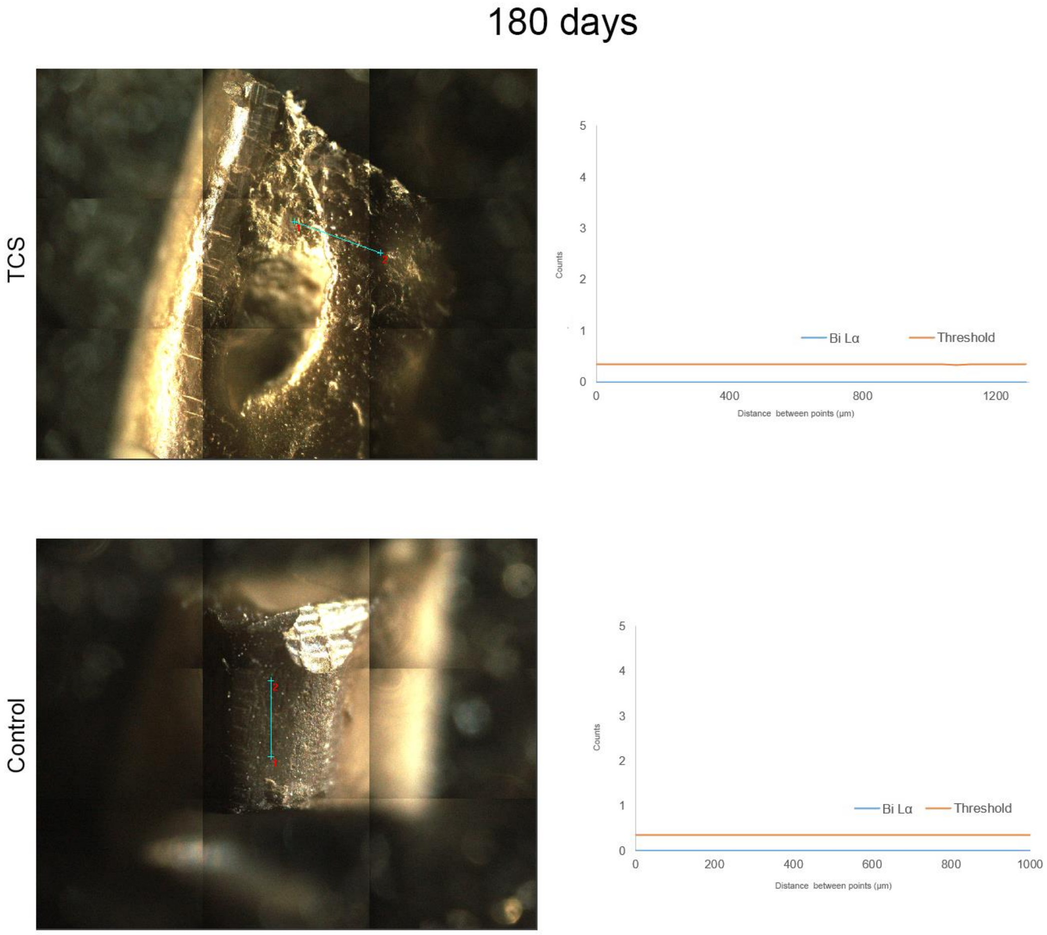

Supplement: Supplementary file 1 — Supplementary Information. [file 41598_2023_36690_MOESM1_ESM.pdf]
